# Color-tunable persistent luminescence in 1D zinc–organic halide microcrystals for single-component white light and temperature-gating optical waveguides†

**DOI:** 10.1039/d2sc01947g

**Published:** 2022-05-25

**Authors:** Bo Zhou, Dongpeng Yan

**Affiliations:** Beijing Key Laboratory of Energy Conversion and Storage Materials, College of Chemistry, and Key Laboratory of Radiopharmaceuticals, Ministry of Education, Beijing Normal University Beijing 100875 P. R. China yandp@bnu.edu.cn

## Abstract

Information security of photonic communications has become an important societal issue and can be greatly improved when photonic signals are propagated through active waveguides with tunable wavelengths in different time and space domains. Moreover, the development of active waveguides that can work efficiently at extreme temperatures is highly desirable but remains a challenge. Herein, we report new types of low-dimensional Zn(ii)–organic halide microcrystals with fluorescence and room-temperature phosphorescence (RTP) dual emission for use as 1D color-tunable active waveguides. Benefiting from strong intermolecular interactions (*i.e.*, hydrogen bonds and π–π interactions), these robust waveguide systems exhibit colorful photonic signals and structural stability at a wide range of extreme simulated temperatures (>300 K), that covers natural conditions on Earth, Mars, and the Moon. Both experimental and theoretical studies demonstrate that the molecular self-assembly can regulate the singlet and triplet excitons to allow thermally assisted spectral separation of fluorescence and RTP, in combination with the single-component standard white-light emission. Therefore, this work demonstrates the first use of metal–organic halide microcrystals as temperature-gating active waveguides with promising implications for high-security information communications and high-resolution micro/nanophotonics.

## Abstract 2

1D zinc–organic halide microcrystals exhibiting thermally assisted spectral separation of fluorescence and phosphorescence could be used as single-component standard white-light and temperature-gating active waveguides.

## Graphical abstract

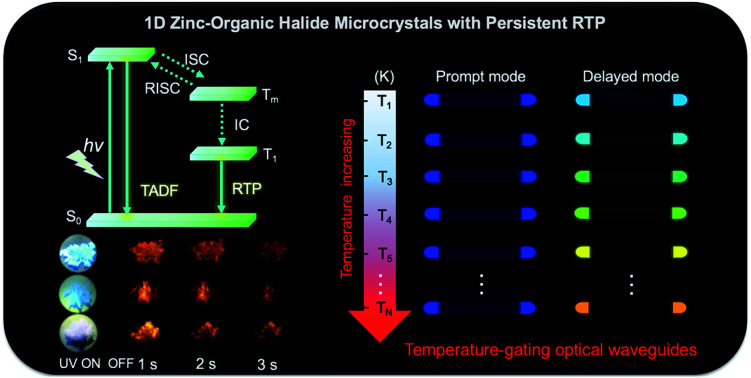

## Introduction

In an era of “knowledge explosion”, information safety during the communication process has become an important issue in human society. Due to the relatively narrow bandwidth, low power density, large energy dissipation, and information leakage potential of traditional electronic devices, researchers have endeavored to construct all-optical devices that can compensate for these drawbacks in electronics.^1–5^ Active waveguides that can transmit photoluminescence (PL) signals from an excited position to the other end have been gradually developed as typical parts of optical devices.^6–9^ To date, most of the propagated PL signals in active waveguides derive from fluorescence, which radiates from singlet excited states with a nanosecond lifetime. In recent years, molecular persistent luminescence (such as room-temperature phosphorescence (RTP) and thermally activated delayed fluorescence (TADF)) with ultralong-lived excited lifetimes has shown great potential in the fields of optical displays and micro/nanophotonics.^10,11^ Moreover, it is worth noting that this persistent luminescence lifetime is usually on the scale of microseconds (ms) to seconds (s) due to the spin-forbidden transition between singlet and triplet excited states, which can be distinguished from fluorescence by time-gating technology.^12–16^ Such difference will be more pronounced if the overlap between prompt fluorescence and decayed emission spectra is decreased. Therefore, it is reasonable to speculate that the security of photonic communication can be greatly improved when the photonic signal is propagated through active waveguides that simultaneously emit fluorescence and phosphorescence with different time and space domains. Particularly, to meet the needs of space travel and future human life, it is imperative to develop active waveguide systems for signal transformation that can work at extremely low/high temperatures; however, no optical waveguide functioning under such wide-range temperature variability has yet been detected in real life.

Metal–organic halide crystalline materials with tunable dimensionality from 3D to 2D, 1D, or 0D have attracted extensive attention in the fields of displays, photocatalysis, photodetectors, and information encryption, among others.^17–28^ Particularly, researchers have demonstrated that metal–organic halides can exhibit high photoluminescence quantum yield (PLQY), which is beneficial to the development of efficient low-dimensional waveguides.^18^ Theoretically, the introduction of heavy atoms (*i.e.*, transition metals and halogens) within metal–organic halides would boost spin–orbit coupling (SOC) and intersystem crossing (ISC) to improve long-lived RTP and/or TADF emission.^29,30^ Furthermore, the construction of rigid metal–organic halide structures with strong intermolecular interactions can suppress non-radiative transitions of triplet excitons, such as thermal vibration and oxygen quenching, to obtain efficient long-persistent luminescence.^31–35^ However, whether it is possible to leverage fluorescence-RTP of metal–organic halides towards color-tunable active waveguides remains a challenge, and such materials with both structural and spectral stability operated at extreme temperatures is still in a speculation.

**Fig. 1:**
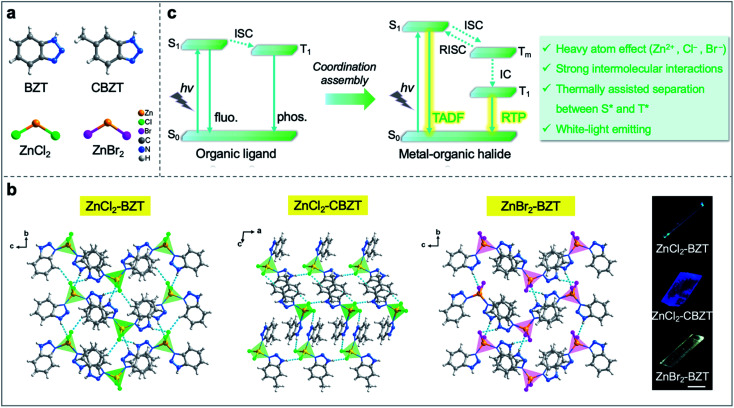
Schematic representation of the tunable singlet and triplet excited states in Zn(ii)-based metal–organic halide microcrystals with active waveguide properties. (a) Related chemical structures. (b) Obtained crystal structures and fluorescence microscopy images of metal–organic halides (ZnCl_2_–BZT, ZnCl_2_–CBZT, and ZnBr_2_–BZT) (scale bar: 100 μm). (c) Comparison of energy level diagrams for organic ligands and metal–organic halides; TADF: thermally activated delayed fluorescence; RTP: room-temperature phosphorescence; RISC: reverse ISC; *T*_m_: intermediate triplet excited states; IC: internal conversion.

Herein, we designed and synthesized a series of low-dimensional Zn(ii)-based metal–organic halide microcrystals (ZnCl_2_–BZT, ZnCl_2_–CBZT, and ZnBr_2_–BZT) with fluorescence and RTP dual emission by employing π-conjugated phosphors (1*H*-benzotriazole, BZT and 5-methyl-1*H*-benzotriazole, CBZT) as organic ligands (Fig. 1a and b). Under unfocused UV irradiation (365 nm), the metal–organic halide microrods or microsheets exhibit typical 1D active waveguide properties, producing bright PL emissions at the end of microcrystals, but weaker emissions within the main body.^36–40^ Due to strong intermolecular interactions (*i.e.*, hydrogen bonds and π–π interactions), these rigid low-dimensional active waveguide systems can maintain their structural stability and integrity at a wide range of simulated extreme temperatures (>300 K), including natural conditions on Earth, Mars, and the Moon. Significantly, the metal–organic halide active waveguides present thermally assisted separation between singlet and triplet excited states, thus avoiding the spectral overlap for fluorescence and RTP, and guaranteeing high resolution and security of information photonic communication. When the halogen ion is replaced by Br^−^ with a strong heavy atom effect in ZnBr_2_–BZT, the singlet and triplet excitons can be further adjusted to achieve white-light emission, which is derived from a pair of perfect complementary colors—blue fluorescence and orange phosphorescence. Detailed time-resolved spectra investigation implies that effective connection between fluorescence and RTP is established by the production of a series of abundant intermediate triplet excited states in these metal–organic halide active waveguide systems (Fig. 1c). Therefore, this work not only demonstrates the ready tuning capacity of singlet and triplet excitons for light-emitting metal–organic halides through molecular self-assembly, but also presents the first proof-of-concept for the use of temperature-gating active waveguide systems in information photonic communication with high resolution and security.

## Results and discussion

### Low-dimensional metal–organic halide microcrystals

Metal–organic halide microcrystals were synthesized *via* a green and facile procedure in aqueous solution. Single-crystal X-ray diffraction analysis reveals that ZnCl_2_–BZT and ZnBr_2_–BZT crystallize in a monoclinic crystal system with the space group of *P*2_1_/*n*, while ZnCl_2_–CBZT crystallizes in a triclinic crystal system with the space group of *P*1̄ (Table S1 and S2†). In these microcrystals, the central Zn^2+^ ion is four-coordinated by two nitrogen atoms from two organic ligands (BTZ or CBZT) and two halogen ions (Cl^−^ or Br^−^) (Fig. S1†). These independent 0D tetrahedral structures are connected *via* hydrogen bonding interactions (N–H⋯X, C–H⋯X) and π–π interactions of organic ligands, endowing the metal–organic halide systems with dense stacking, potentially inhibiting the non-radiative decay of triplet excitons, and improving the thermal stability (Table S3†). Compared with the pristine organic ligands BZT and CBZT, the FT-IR spectra of metal–organic halides present intensified and red-shifted vibrational peaks of N–H around 3160 cm^−1^, which confirm the formation of strong hydrogen bonding interactions (Fig. S2†). Moreover, according to thermal gravimetric analysis (TGA), metal–organic halide microcrystals exhibit good thermal stability with a decomposition temperature up to 501 K (Fig. S3†). The rigid and stable structures of metal–organic halide microcrystals are critical prerequisites to the development of active waveguide systems that can work at extreme temperatures.

### Tunable fluorescence and RTP of the metal–organic halide microcrystals

**Fig. 2:**
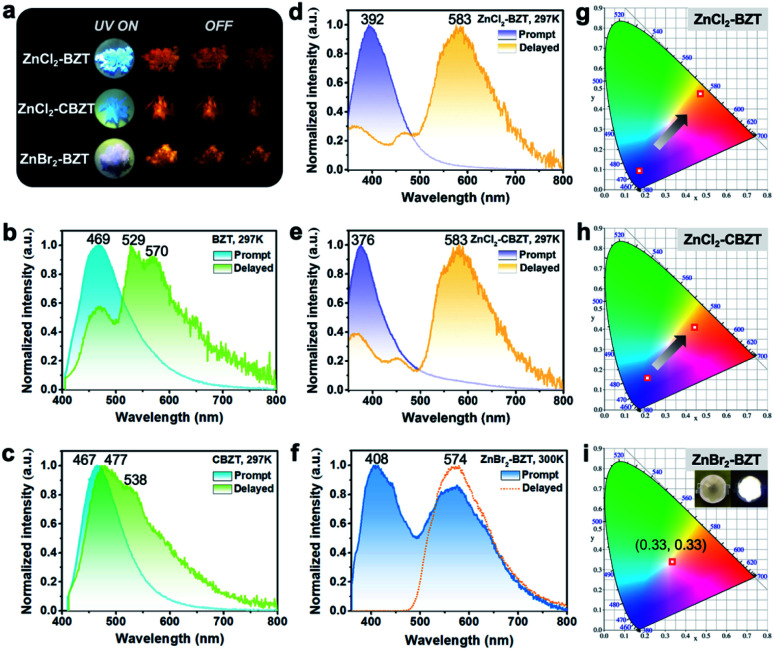
Tunable fluorescence and RTP of the metal–organic halide microcrystals. (a) Photographs of the luminescent metal–organic halide microcrystals before and after cessation of the 365 nm irradiation. Prompt and delayed (acquired after 0.01 ms of excitation) PL spectra for the organic ligands BZT (b), CBZT (c), ZnCl_2_–BZT (d), ZnCl_2_–CBZT (e), and ZnBr_2_–BZT (f). Corresponding positions in CIE chromaticity coordinates for metal–organic halide microcrystals under prompt and delayed modes (g, h and i), respectively.

Under UV light excitation at 365 nm, the metal–organic halides ZnCl_2_–BZT, ZnCl_2_–CBZT and ZnBr_2_–BZT exhibit pale blue, blue and white emissions, respectively. Upon cessation of excitation, all emission colors change to orange and persist for at least 2 s (Fig. 2a). Owing to the large differences in emission colors before and after cessation of UV irradiation, short-lived (UN ON) and long-lived (UV OFF) emissions are easily distinguishable by the naked eye. Based on the spectral studies, the prompt and delayed emission positions of the pristine ligands BZT (469 nm and 529 nm) and CBZT (467 nm and 477 nm) are close to each other, indicating incomplete separation between fluorescence and phosphorescence (Fig. 2b and c and S4†). However, through effective coordination self-assembly, the metal–organic halides ZnCl_2_–BZT (392 nm and 583 nm) and ZnCl_2_–CBZT (376 nm and 583 nm) exhibit a strong ability to separate prompt and delayed spectra (Fig. 2d, e, g and h). Impressively, when the halogen ion Cl^−^ is changed to Br^−^ with a stronger heavy atom effect, the prompt (408 nm) and delayed (574 nm) spectra of ZnBr_2_–BZT can be further adjusted to allow standard white-light emission with the chromaticity coordinate of (0.33, 0.33) (Fig. 2f and i). The absolute PLQY values of ZnCl_2_–BZT, ZnCl_2_–CBZT, and ZnBr_2_–BZT are 13.01%, 5.43%, and 8.01%, respectively. The above observations indicate the positions and intensities of singlet and triplet excitons can be highly adjusted by modulation of both the coordination self-assembly units and intermolecular interactions.

**Fig. 3:**
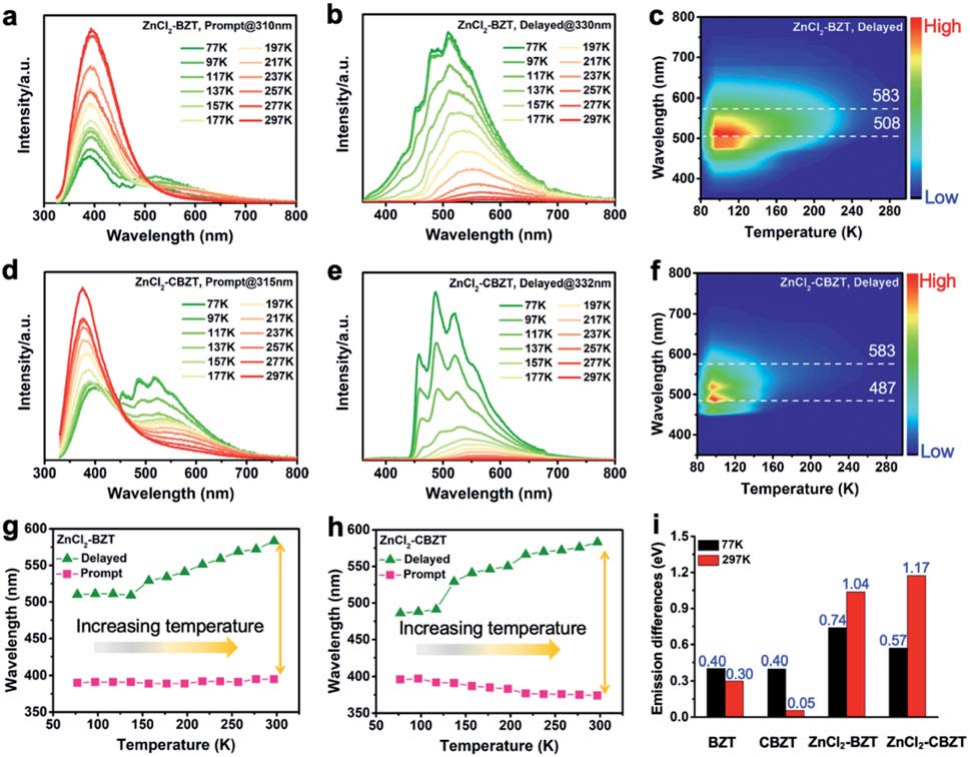
Temperature-dependent spectra and time-resolved decay measurements. Prompt and delayed spectra of ZnCl_2_–BZT (a, b and c) and ZnCl_2_–CBZT (d, e and f) at different temperatures ranging from 77 K to 297 K. Comparison of dominant emission positions of ZnCl_2_–BZT (g) and ZnCl_2_–CBZT (h) under prompt and delayed modes. (i) Calculated differences between dominant emission peaks under prompt and delayed modes for BZT, CBZT, ZnCl_2_–BZT, and ZnCl_2_–CBZT at 77 K and 297 K.

To investigate the photophysical processes of metal–organic halides, a series of temperature-dependent spectra and time-resolved decay measurements were systematically performed. Temperature-dependent prompt spectra show that the emission intensities of ZnCl_2_–BZT at 392 nm, ZnCl_2_–CBZT at 376 nm, and ZnBr_2_–BZT at 408 nm gradually increase as temperature rises, suggesting that the energy levels at 392 nm, 376 nm and 408 nm are thermally activated (Fig. 3a and d and S5a†). According to the time-resolved decays, the main emission peaks of these prompt spectra simultaneously display short-lived (nanosecond scale) and long-lived (microsecond scale) lifetimes, proving that these emissions feature typical TADF characteristics through the reverse ISC (RISC) process between triplet and singlet excited states (Fig. S6†). The thermal activation energies between excited singlet and triplet states (Δ*E*_st_) can be determined as 0.17 eV (ZnCl_2_–BZT), 0.27 eV (ZnCl_2_–CBZT), and 0.059 eV (ZnBr_2_–BZT) based on eqn (1), which are confirmed to be small enough to realize the RISC process (Fig. S7a, S7c and S7e†).^46,47^Eqn (1) is used to quantitatively evaluate temperature-dependent changes in prompt and delayed emissions:1
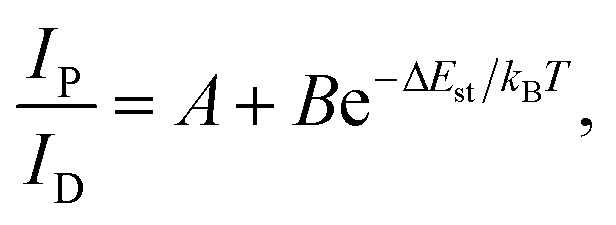
in which *I*_P_/*I*_D_ refers to the integrated intensity ratio between prompt and delayed spectra, and *k*_B_ is the Boltzmann constant. With increasing temperature, the emission intensities of delayed spectra for ZnCl_2_–BZT, ZnCl_2_–CBZT and ZnBr_2_–BZT gradually decrease, and the corresponding emission peaks appear red-shifted, which could be attributed to the increased non-radiative transitions and stronger vibrational coupling at higher temperatures (Fig. 3b, c, e and f and S5b†). In addition, the delayed lifetimes of the dominant emission peaks for three metal–organic halide microcrystals decrease greatly as temperature rises from 77 K to 297 K (Fig. S8 and Table S4†). Therefore, the dominant emissions for ZnCl_2_–BZT (583 nm, 0.68 ms), ZnCl_2_–CBZT (583 nm, 1.84 ms), and ZnBr_2_–BZT (574 nm, 22.10 ms) in delayed spectra at 297 K can be ascribed to RTP derived from triplet excitons.^56^ The binding energy (*E*_b_) of triplet excitons can be estimated through Arrhenius plotting of integrated intensity of the delayed spectra *versus* the reciprocal of temperature according to the following equation:2
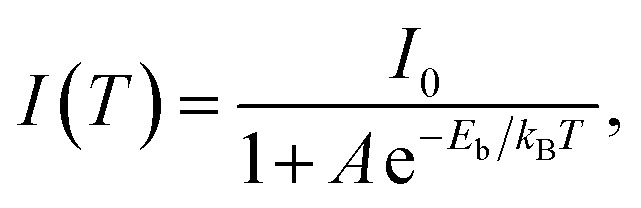
in which *I*_0_ refers to the integrated intensity of delayed spectra at 0 K. According to this equation, *E*_b_ of ZnCl_2_–BZT, ZnCl_2_–CBZT, and ZnBr_2_–BZT can be calculated as 0.079 eV, 0.055 eV, and 0.13 eV, respectively, all of which exceed the thermal energy (0.026 eV), and are therefore conducive to the radiative recombination of triplet excitons (Fig. S7b, S7d and S7f†).^17^

Typically, the dominant emission peaks of prompt and delayed spectra for ZnCl_2_–BZT and ZnCl_2_–CBZT gradually separate from one another when the temperature is increased (Fig. 3g and h). The differences in dominant emission peaks under prompt and delayed modes increase from 0.74 eV to 1.04 eV for ZnCl_2_–BZT and 0.57 eV to 1.17 eV for ZnCl_2_–CBZT when the temperature rises from 77 K to 297 K, generating values much higher than those of pristine BZT (77 K: 0.40 eV; 297 K: 0.30 eV) and CBZT (77 K: 0.40 eV; 297 K: 0.05 eV) (Fig. 2, 3i and S9†). Apparently, these emission differences between prompt and delayed modes of BZT and CBZT decrease when the temperature increases from 77 K to 297 K. Considering that ZnBr_2_–BZT has a strong heavy atom effect, the enhanced SOC process can greatly promote ISC from the singlet to triplet excited state; thus, the emissions of prompt spectrum are dominated by phosphorescence in a wide temperature range which is overlapped by the corresponding delayed spectrum (Fig. S5†). As the phosphorescence of ZnBr_2_–BZT exhibits a gradual red shift with increasing temperature, white-light emission can be ultimately realized by a combination of the highly separated fluorescence (408 nm) and RTP (574 nm). Therefore, due to the tunable heavy atom effect, the metal–organic halides can not only exhibit thermally assisted separation of fluorescence and RTP, but also can support standard white-light emission with the CIE chromaticity coordinate of (0.33, 0.33) at room temperature, an ability that remains rather rare among state-of-the-art single-component systems.^48–50,57^

### Mechanism of the light-emitting metal–organic halide microcrystals

**Fig. 4:**
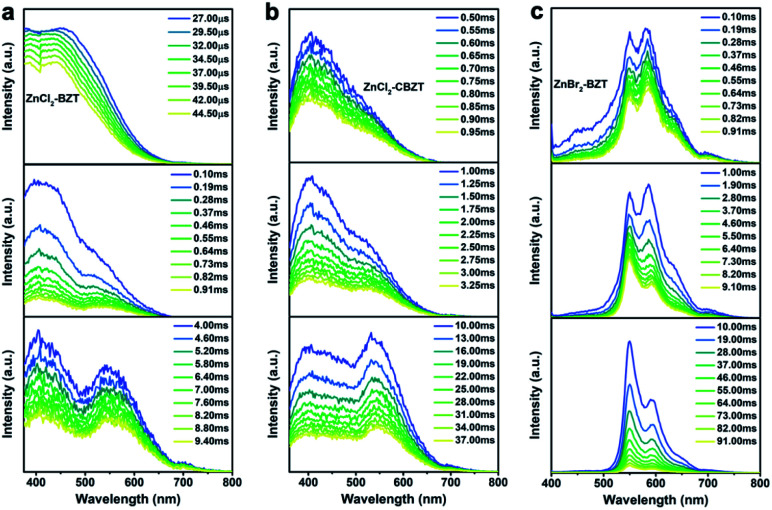
Detecting the intermediate triplet excited states. Time-resolved emission spectra (TRES) of ZnCl_2_–BZT (a), ZnCl_2_–CBZT (b), and ZnBr_2_–BZT (c), which indicate that the energy of triplet excitons can be transferred from higher energy levels (400 nm–500 nm) to lower energy levels (500 nm–575 nm) through a rapid IC process which is followed by emission from the lowest triplet excited states.

Of note, the energy gaps between dominant fluorescence (singlet excited state) and phosphorescence (triplet excited state) at 297 K for ZnCl_2_–BZT (1.04 eV), ZnCl_2_–CBZT (1.17 eV), and ZnBr_2_–BZT (0.88 eV) are relatively large, which is seemingly difficult to achieve with direct ISC or RISC.^46,47^ Given that the energy levels of singlet excited states for these metal–organic halides are almost constant from 77 K to 297 K, whereas the triplet excited states exhibit a significant red-shift with increased temperature, intermediate triplet excited states should be appreciated as “bridges” to link dominant fluorescence and phosphorescence. To detect the possibility of intermediate triplet excited states, a systematic series of time-resolved emission spectra (TRES) in the microsecond or millisecond scale were collected. As depicted in Fig. 4, relative emission intensities gradually decrease in the wavelength range of 400 nm to 500 nm for ZnCl_2_–BZT, ZnCl_2_–CBZT, and ZnBr_2_–BZT, while these values increase with time in the wavelength range from 500 nm to 575 nm.^41^ Therefore, the TRES experiment indicates that the energy of triplet excitons can be transferred from higher energy levels (400 nm–500 nm) to lower energy levels (500 nm–575 nm) through a rapid IC process, with emission from the lowest triplet excited states ultimately generating the dominant phosphorescence. Furthermore, excitation peaks range from 400 nm to 525 nm in the delayed excitation spectra, which overlap with the observed energy levels of intermediate triplet excited states in TRES, and thus elucidate that these intermediate triplet excited states are highly excitable (Fig. S10†). Consequently, it can be concluded that effective connections between dominant fluorescence and phosphorescence across large energy gaps are established by the production of a series of abundant intermediate triplet excited states that serve as “bridges”.^51–55^

In order to gain more insight into the luminescent nature of the metal–organic halide microcrystals, we recorded prompt and delayed spectra of the solutions of the pristine ligands BZT and CBZT at 77 K (Fig. S11†). It is apparent that BZT and CBZT exhibit fluorescence–phosphorescence dual emission with energy gaps reaching 1.14 eV and 1.10 eV, respectively, analogous to the highly spectral separation observed for metal–organic halides at 297 K (Fig. 3i). Moreover, these detected intermediate triplet excited states (400 nm–500 nm, 500 nm–575 nm) of metal–organic halides can be observed in the cryogenic spectra of organic ligand solutions, and the triplet excited state levels of the metal–organic halides at 583 nm remain detectable in the delayed spectra of BZT and CBZT solutions. As the central Zn^2+^ ion with the d^10^ electronic configuration lacks luminescence properties, the organic ligands BZT and CBZT are deemed as the core luminescent components of these light-emitting metal–organic halides. However, serious non-radiative transitions of triplet excitons engender phosphorescence quenching of organic ligands solutions at room temperature. In contrast, benefiting from the rigid structures of metal–organic halides through the effective coordination assembly, non-radiative transitions of triplet excitons can be effectively suppressed to allow ultralong phosphorescence at room temperature, easily realizing high separation between fluorescence and phosphorescence in metal–organic halides under ambient temperature and atmosphere conditions.

**Fig. 5:**
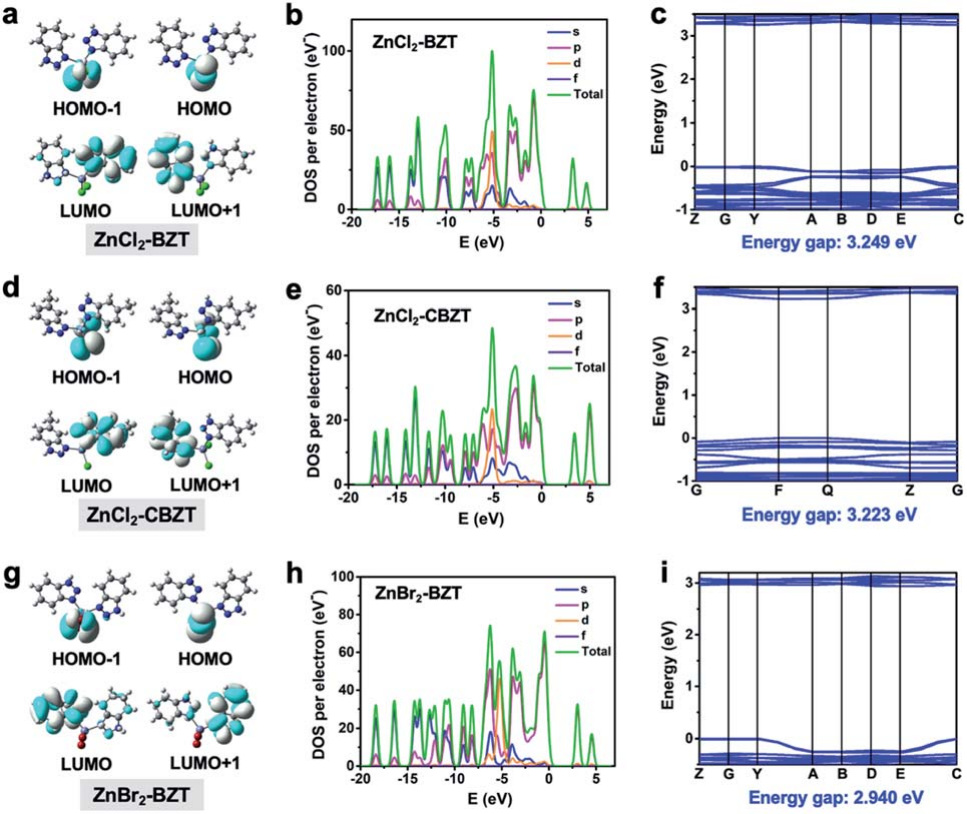
Determining different roles of organic ligands and halogen ions in luminescence behaviors. Calculated molecular orbitals, the band structures around Fermi energy levels and the total/partial electronic density of states for ZnCl_2_–BZT (a, b and c), ZnCl_2_–CBZT (d, e and f), and ZnBr_2_–BZT (g, h and i).

While organic ligands determine the occurrence of fluorescence and phosphorescence in metal–organic halide microcrystals, halogen ions can be used to further adjust energy levels to achieve different emission behaviors. To assess the influence of halogen ions on luminescence, electron-density distributions, density of states (DOS), band structures, and electrostatic potentials (ESP) were calculated by using periodic density functional theory (DFT). Frontier orbital analyses indicate that the highest occupied molecular orbital (HOMO) and the lowest unoccupied molecular orbital (LUMO) are localized on halogen ions (Cl^−^ or Br^−^) and organic ligands (BTZ or CBZT), respectively, suggesting the occurrence of the halogen-to-ligand charge transfer (XLCT) process (Fig. 5a, d and g). This XLCT process can also be corroborated by the solid-state UV-vis absorption spectra, which exhibit a broad absorption band from 450 nm to 600 nm for metal–organic halides compared with the pristine ligands (Fig. S12†). The total electronic density of states (TDOS) and partial electronic density of states (PDOS) demonstrate that the valence bands (VB) originate from the p orbitals of halogen ions, while the conduction bands (CB) derive from the p orbitals of C and N atoms (Fig. 5b, e and h and S13–S15†). Thereafter, all theoretical calculations not only confirm that the organic ligands BZT and CBZT are the core luminescent components of metal–organic halide microcrystals, but also prove that halogen ions participate in the luminescence of metal–organic halides. The halogen Cl, which has greater electronegativity, can stabilize the halide-based VB more than the halogen Br; thus the calculated band gaps of ZnCl_2_–BZT (3.249 eV) and ZnCl_2_–CBZT (3.223 eV) are greater than that of ZnBr_2_–BZT (2.940 eV) (Fig. 5c, f and i).^42,43^ These band gap calculations are consistent with experimental spectral results, in which emissions from singlet excited states of ZnCl_2_–BZT (3.18 eV, 392 nm) and ZnCl_2_–CBZT (3.31 eV, 376 nm) appear blue-shifted compared with that of ZnBr_2_–BZT (3.05 eV, 408 nm). Moreover, ESP calculations reveal the respective electron density distributions (red color area: higher electron density; blue color area: lower electron density), which highlight the trend in charge transfer from halogen ions to organic ligands (Fig. S16 and Table S5†). Net ESP values between the halogen ions (electron donor) and organic ligands (electron acceptor) in ZnCl_2_–BZT, ZnCl_2_–CBZT, and ZnBr_2_–BZT are calculated to be −21.51 kcal mol^−1^, −70.26 kcal mol^−1^, and −14.32 kcal mol^−1^, respectively. Among the three metal–organic halides, ZnBr_2_–BZT shows the highest net value of ESP, indicating that the halogen ion Br^−^ is most inclined to donate the electron, causing a red-shift of the fluorescence emission of ZnBr_2_–BZT. Therefore, through effective halogen regulation, the prepared metal–organic halide microcrystals can further generate the single-component white-light emission for ZnBr_2_–BZT.

### Temperature-gating low-dimensional active waveguides and photonic communications

**Fig. 6:**
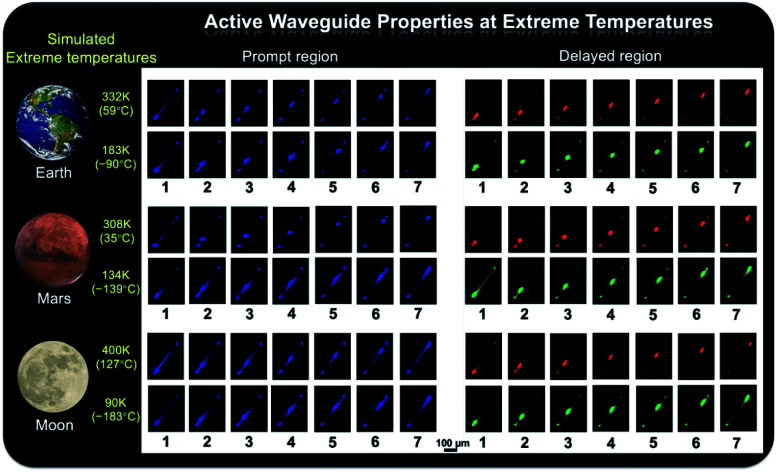
Active waveguide properties at extreme temperatures. PL microscopy images of 1D microrod ZnCl_2_–BZT obtained by 375 nm laser excitation at different local positions under simulated extreme surface temperatures of (332 K and 183 K), Mars (308 K and 134 K), and the Moon (400 K and 90 K). Prompt regions: 420 nm–500 nm; delayed regions: 550 nm–600 nm (at high temperature), 500 nm–550 nm (at low temperature).

As shown in Fig. 1b, the prepared metal–organic halide microcrystals exhibit typical 1D active waveguide properties under unfocused UV irradiation. According to the differential scanning calorimetry (DSC) measurements, the crystal structures of active metal–organic halide waveguides are highly stable in the temperature range of 83 K to 463 K, covering the extreme surface temperatures of Earth, Mars, and the Moon (Fig. S17†). Subsequently, we employed 1D microrod ZnCl_2_–BZT as a model system to simulate active waveguide performance under extreme temperatures. Detailed spatially resolved PL microscopy images were obtained under 375 nm laser excitation at different local positions on this 1D microrod ZnCl_2_–BZT. Due to the rigid microstructure of ZnCl_2_–BZT, the generated photon signals are confined and transmitted along the 1D microrod structure without significant optical signal loss. Active waveguide performance can be evaluated by calculating the optical-loss coefficient (*R*) according to the exponential decay formula *I*_tip_/*I*_body_ = *A* exp(−*RD*). In this formula, *I*_tip_ and *I*_body_ represent the emission intensities of the emitting tip and excited positions, respectively, *A* refers to the ratio of energy escaping and propagating, and *D* is the propagation distance between the emitting tip and excited position. By detecting and collecting emission signals in the prompt (420 nm–500 nm) and delayed regions (at high temperature: 550 nm–600 nm; at low temperature: 500 nm–550 nm), microrod ZnCl_2_–BZT exhibits significant 1D active waveguide capacity at the simulated extreme surface temperatures of Earth (332 K and 183 K), Mars (308 K and 134 K), and the Moon (400 K and 90 K) (Fig. 6). The optimized optical loss coefficient *R* is determined to be 6.2 × 10^−4^ dB mm^−1^ (Fig. S18 and Table S6†), notably lower than those of state-of-the-art reported molecular waveguide materials.^7,44,45,58^

**Fig. 7:**
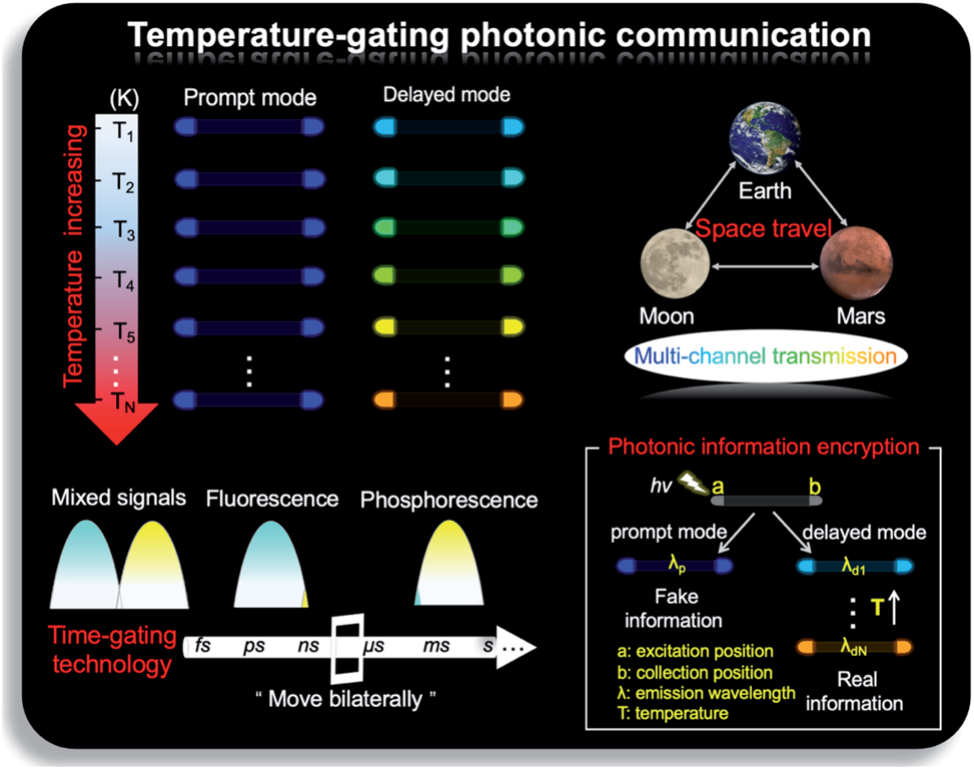
Temperature-gating photonic communication. Diagram depicting the use of a 1D active waveguide microrod ZnCl_2_–BZT in the field of temperature-gating photonic communications, which could transmit fluorescence and phosphorescence signals with different time and space domains over a large temperature span, enabling an improved density and high security of photonic communication.

After assessing the 1D active waveguide microrod ZnCl_2_–BZT with high thermostability, we endeavored to demonstrate the temperature-gating photonic communication with high resolution and security level. Of note, the phosphorescence exhibits obvious differences in wavelengths and colors between high and low temperatures (Fig. 3g and h); thus, temperature-dependent multi-channel photonic signals can be conveyed within the individual 1D microrod, significantly enhancing information storage and transmission capability. Such temperature-dependent colored waveguides can also serve as alternatives to the widely used wavelength division multiplexing (WDM) in fiber communications. Moreover, to the best of our knowledge, this work represents the first successful report of active optical waveguide systems operating in such a wide temperature range (from 90 K to 400 K) based on fluorescence and phosphorescence dual emission,^36,37^ which provides a major opportunity for the application of information photonic communication to future astronomical exploration, not only on Earth, but also on terrestrial planets (such as Mars) and satellites (such as the Moon). Based on the analyses above, fluorescence and phosphorescence predominate in the prompt and delayed emission regions, respectively (Fig. S19†). As depicted in Fig. 7, in contrast to fluorescence (prompt mode) with nearly unchanged emission, the phosphorescence (delayed mode) of 1D metal–organic halide microrods is temperature-sensitive, which is conducive to the development of temperature-gating multi-channel transmission methods for photonic signals and supports greatly improved information transmission capacity. Fluorescence and phosphorescence signals at each temperature can be easily distinguished by time-gating technology.^59,60^ Due to the high thermostability of these synthesized active waveguide microrods, photonic signals could someday be transmitted between Earth, Mars, and the Moon. Moreover, the development of a 1D active waveguide microrod can serve as an advanced temperature-gating information photonic encoding system, particularly applicable at various extreme temperatures. As a demo, we constructed temperature-gating 1D active waveguide microrods to transmit encrypted photonic information (phosphorescence) at a specific temperature. Due to the time-resolved properties of fluorescence (nanoseconds) and phosphorescence (microseconds), the receiving end must process these photonic signals under delayed mode to obtain the real information (phosphorescence signals). Therefore, the 1D active waveguide microrod ZnCl_2_–BZT can be used to transmit fluorescence and phosphorescence signals with different time and space domains over a large temperature span, which enables an improved density and high security of photonic communication.

## Conclusions

In summary, low-dimensional Zn(ii)-based metal–organic halide microcrystals (ZnCl_2_–BZT, ZnCl_2_–CBZT, and ZnBr_2_–BZT) can be facilely fabricated *via* a coordination self-assembly process, enabling highly tunable persistent luminescence, with highly separated fluorescence and phosphorescence. Through effective halogen regulation, the metal–organic halides can not only allow fluorescence–phosphorescence dual emission with different time-space domains, but also support single-component standard white-light emission for ZnBr_2_–BZT. Detailed time-resolved spectra investigation proves that the produced abundant intermediate triplet excited states serve as “bridges” to effectively link fluorescence and RTP across large energy gaps. Impressively, these metal–organic halide microcrystals exhibit 1D active waveguide properties with an optimized optical loss coefficient of 6.2 × 10^−4^ dB mm^−1^, much lower than those of most state-of-the-art molecule-based waveguides. These robust low-dimensional active waveguides exhibit color-tunable persistent luminescence and high structural stability under the simulated extreme conditions relevant to Earth, Mars, and the Moon. Such systems largely improve the information security level and signal transfer capability relative to the typical fluorescent waveguides. Therefore, this work provides an effective way to construct temperature-gating low-dimensional active waveguides across a wide measurement range (>300 K), significantly extending the potential of light-emitting metal–organic halides in the fields of information security and photonic communication for future space exploration and astronomical technology development.

## Data availability

Experimental procedures, details of the calculations, and additional data can be found in the ESI.†

## Author contributions

Bo Zhou and Dongpeng Yan conceived the experiments. Bo Zhou conducted and analyzed the experiments. Dongpeng Yan supervised the project. All authors prepared and edited the manuscript.

## Conflicts of interest

There are no conflicts to declare.

## Supplementary Material

SC-013-D2SC01947G-s001

SC-013-D2SC01947G-s002
