# Supplementary material for: Color-tunable persistent luminescence in 1D zinc–organic halide microcrystals for single-component white light and temperature-gating optical waveguides
Source: Chem Sci. 2022 May 25;13(25):7429–36. doi: 10.1039/d2sc01947g (PMC9242015; doi:10.1039/d2sc01947g)
Supplement: SC-013-D2SC01947G-s001 [file SC-013-D2SC01947G-s001.pdf]

Electronic Supplementary Material (ESI) for Chemical Science.
This journal is © The Royal Society of Chemistry 2022

Electronic Supplementary Information

Color-tunable Persistent Luminescence in 1D Zinc-organic Halide Microcrystals for Single-component White Light and Temperature-gating Optical Waveguides

*Bo Zhou and Dongpeng Yan**

Beijing Key Laboratory of Energy Conversion and Storage Materials, College of Chemistry, and Key Laboratory of Radiopharmaceuticals, Ministry of Education, Beijing Normal University, Beijing 100875, P. R. China

*Corresponding Author: yandp@bnu.edu.cn

1. Experimental Section

Materials

All the reagents (1H-benzotriazole, 5-methyl-1H-benzotriazole, zinc chloride, zinc bromide, hydrochloric acid and hydrobromic acid) were purchased from the Sigma Chemistry Co. Ltd. and used without further purification. Distilled water was prepared in our lab.

Characterizations

Single-crystal X-ray diffraction data of these samples were carried out on Rigaku Oxford Diffraction Supernova X-ray source diffractometer equipped with monochromatized Cu-Kα radiation (λ = 1.5406 Å) at room temperature. FT-IR spectra were recorded in the range of 3750−1000 cm^{-1} on a Tensor 27 OPUS (Bruker) FT-IR spectrometer. Solid UV−vis absorption spectra were collected on a Shimadzu UV-3600 spectrophotometer at room temperature. Data were recorded in the wavelength range of 250−800 nm, and $BaSO_4$ powder was used as a standard (100% reflectance). TGA tests were collected on a Perkin-Elmer Diamond SII thermal analyzer under the atmosphere of nitrogen with a heating rate of 10 K min^{-1}. DSC thermograms were measured on the METTLER TOLEDO DSC 1 calorimeter under nitrogen with a heating (or cooling) rate of 10 °C min^{-1}. All the relevant photoluminescence (PL) tests and time-resolved lifetime were conducted on an Edinburgh FLS980 fluorescence spectrometer. The PLQY values of zinc-organic halide microcrystals were recorded by using a Teflon-lined integrating sphere (F-M101, Edinburgh, diameter: 150 mm and weight: 2 kg) accessory in FLS980 fluorescence spectrometer.

Fluorescence microscopic images of crystals were taken under OLYMPUS IXTI fluorescence microscope. Photographs for the afterglow images were captured under iphone X. The ISS Q2 FLIM/FFS confocal system (ISS Inc.) was used to acquire the PL images. The system was attached to a Nikon inverted microscope, equipped with the Nikon 4X/0.2 NA objective lens. Diode lasers of 375 nm was used for the excitation of $ZnCl_2$-BZT. Temperature was controlled by linkmn hot stage with a heating (or cooling) rate of 10 K min^{-1}. The images were acquired using CMOS detector from

TUCSEN (model MIchrome 6) and Mosaic V2.1 software.

Electronic structure calculations of $ZnCl_2$-BZT, $ZnCl_2$-CBZT, and $ZnBr_2$-BZT were performed with the periodic density functional theory (DFT) method by using Dmol3 module in Material Studio software package.[1,2] The initial configurations were fully optimized by the Perdew-Wang (PW91) generalized gradient approximation (GGA) method with the double numerical basis sets plus polarization function (DNP).[3] The core electrons of metals were treated by effective core potentials (ECP). The self-consistent field (SCF) converged criterion was within 1.0×10^{-5} hartree per atom and the converged criterion of the structure optimization was 1.0×10^{-3} hartree per bohr. The Brillouin zone was sampled by $1 \times 1 \times 1$ *k*-points, and calculation tests reveal that the increase in *k*-points does not affect the results. Gaussian 09 program [4] was used and the geometric optimization of the compound was carried out with the 6-31G+ basis set and B3LYP functional which were chosen for the high-quality theoretical approach.[5–7] The wavefunction files for the compound were also obtained from the Gaussian 09W, which serves as the input file for multiwfn 3.4.1, a wavefunction analyser software. The analyses for electrostatic potential were finished by Multiwfn 3.4.1,[8] which is a multifunctional wavefunction analysis program. All isosurface maps were rendered by VMD 1.9.1 program [9] based on the outputs of Multiwfn. During the quantitative analyses of electrostatic potential on van der Waals (vdW) surface in Multiwfn program, the grid spacings were set to 0.25 Bohr.

Synthesis of Zinc-Organic Halide Microcrystals

$ZnCl_2$-BZT, $ZnCl_2$-CBZT or $ZnBr_2$-BZT was prepared by mixing the 1H-benzotriazole (119.1 mg, 1 mmol) or 5-methyl-1H-benzotriazole (133.1 mg, 1 mmol), zinc chloride (136.3 mg, 1 mmol) or zinc bromide (225.2 mg, 1 mmol), hydrochloric acid (20 μL, 36%) or hydrobromic acid (40 μL, 48%), and distilled water (5 mL) as starting materials in the reaction vials. After being evaporated at 45 °C for 72 hours, transparent microcrystals were obtained, with the reaction yield up to 87% for ZnCl2-BZT, 81% for ZnCl2-CBZT, and 90% for ZnBr2-BZT, respectively. Hydrochloric acid and hydrobromic acid are used to facilitate the formation of metal-organic halide

microcrystals. [CCDC 2108186, 2108187 and 2108189 contain the supplementary crystallographic data for this paper. These data can be obtained free of charge from The Cambridge Crystallographic Data Centre]

2. Structural characterization and thermal stability

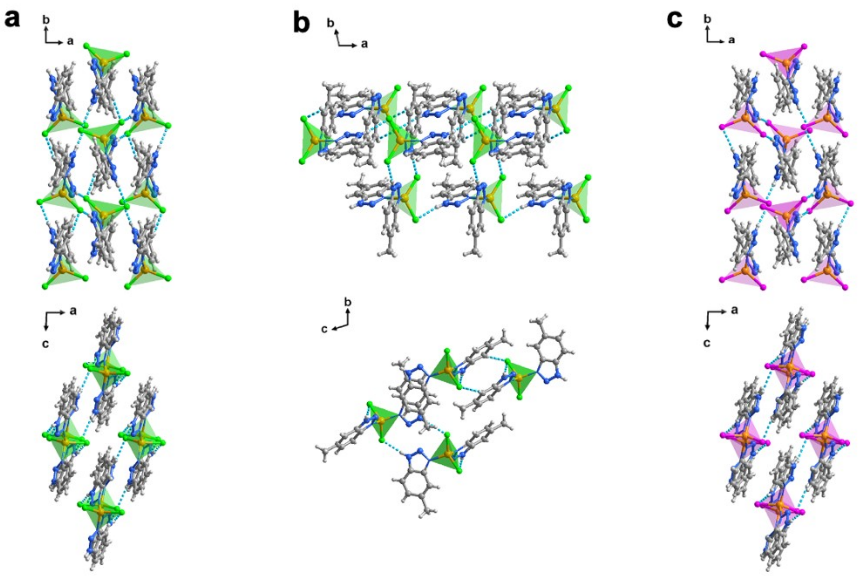

Fig. S1 The crystal structures of $ZnCl_2$-BZT (a), $ZnCl_2$-CBZT (b), and $ZnBr_2$-BZT (c).

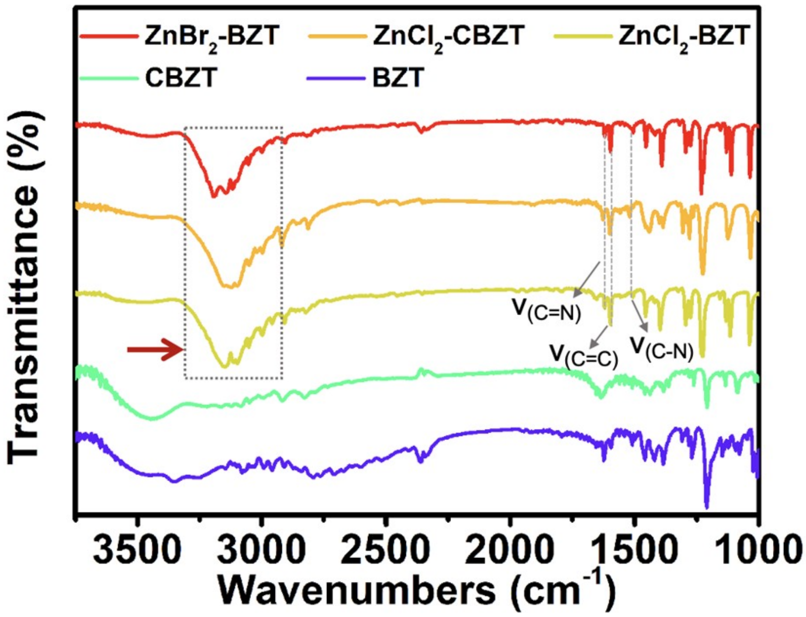

Fig. S2 FT-IR spectra of metal-organic halides present intensified and red-shifted vibrational peaks of N-H around 3160 cm^{-1}, which confirm the formation of strong hydrogen bonding interactions. $V_{(C=N)}$: 1621 cm^{-1}; $V_{(C=C)}$: 1600 cm^{-1}; $V_{(C\text{-}N)}$: 1517 cm^{-1}.

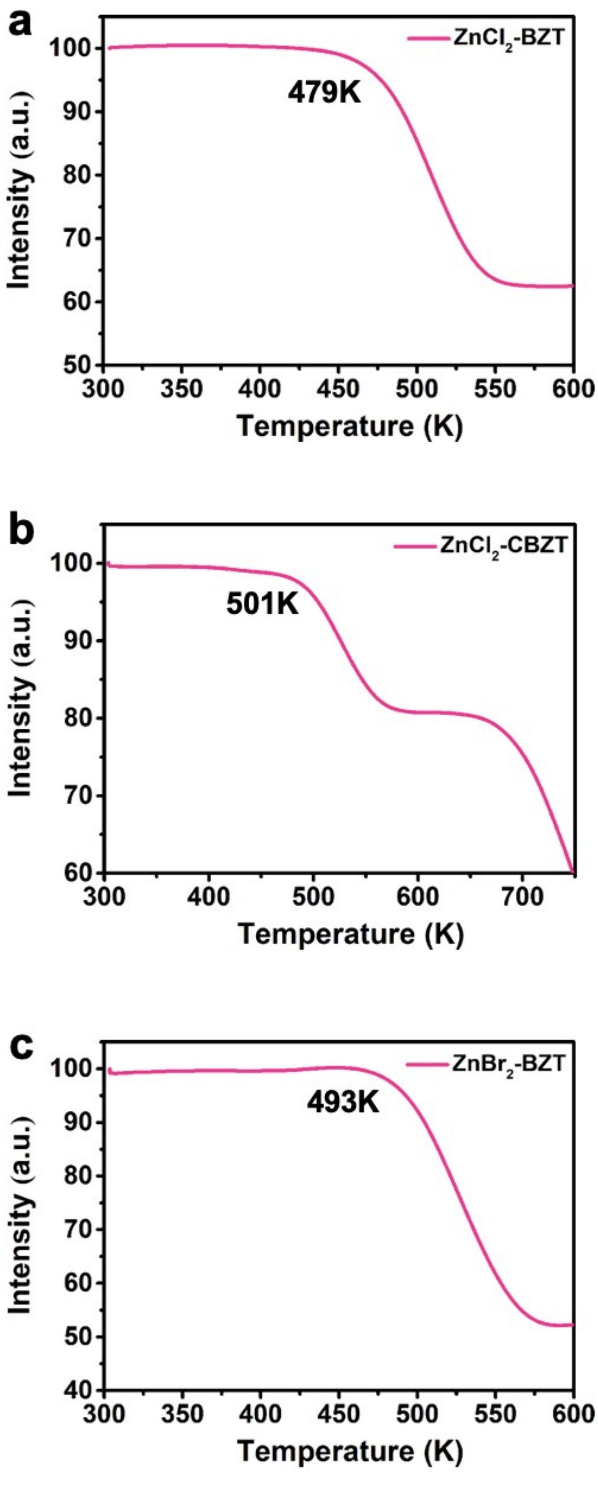

Fig. S3 The TGA curves of $ZnCl_2$-BZT (a), $ZnCl_2$-CBZT (b), and $ZnBr_2$-BZT (c).

Table S1. Crystallographic data collection and refinement details for metal-organic halide microcrystals $ZnCl_2$-BZT, $ZnCl_2$-CBZT and $ZnBr_2$-BZT at 100 K.

Metal complexes	$ZnCl_2$-BZT	$ZnCl_2$-CBZT	$ZnBr_2$-BZT
Molecular Formula	$C_{12}H_{10}Cl_2N_6Zn$	$C_{14}H_{14}Cl_2N_6Zn$	$C_{12}H_{10}Br_2N_6Zn$
Molecular Weight	374.53	399.59	463.45
Temperature (K)	100	100	100
Crystal System	Monoclinic	Triclinic	Monoclinic
Space Group	$P2_1/n$	P-1	$P2_1/n$
a (Å)	7.5599	7.5306	7.8171
b (Å)	13.9242	8.4502	14.4787
c (Å)	13.8315	14.2634	13.5772
α (deg)	90	106.449	90
β (deg)	92.303	92.468	94.249
γ (deg)	90	101.093	90
V ($Å^3$)	1454.80	849.64	1532.46
Z	4	2	4
Density (g/cm^3)	1.710	1.562	2.009
GooF	1.039	1.108	1.101
R [$I > 2\sigma(I)$]	R_1 = 0.0245, wR_2 = 0.0661	R_1 = 0.0617, wR_2 = 0.1783	R_1 = 0.0211, wR_2 = 0.0540
CCDC number	2108187	2108189	2108186

Table S2. Crystallographic data collection and refinement details for metal-organic halide microcrystals $ZnCl_2$-BZT, $ZnCl_2$-CBZT and $ZnBr_2$-BZT at 297 K.

Metal complexes	$ZnCl_2$-BZT	$ZnCl_2$-CBZT	$ZnBr_2$-BZT
Molecular Formula	$C_{12}H_{10}Cl_2N_6Zn$	$C_{14}H_{14}Cl_2N_6Zn$	$C_{12}H_{10}Br_2N_6Zn$
Molecular Weight	374.53	399.59	463.45
Temperature (K)	297	297	297
Crystal System	Monoclinic	Triclinic	Monoclinic
Space Group	$P2_1/n$	P-1	$P2_1/n$
a (Å)	7.7541	7.5887	7.9567
b (Å)	13.9872	8.4931	14.4098
c (Å)	13.7990	14.4611	13.7541
α (deg)	90	106.299	90
β (deg)	92.5329	91.949	94.419
γ (deg)	90	101.128	90
V ($Å^3$)	1495.15	873.91	1572.28
Z	4	2	4
Density (g/cm^3)	1.664	1.530	1.958
GooF	1.278	1.084	1.066
R [$I > 2\sigma(I)$]	R_1 = 0.0482, wR_2 = 0.1450	R_1 = 0.0573, wR_2 = 0.1764	R_1 = 0.0302, wR_2 = 0.0777
CCDC number	2108191	2108188	2108190

Table S3. The hydrogen bonds and π–π interactions of $ZnCl_2$-BZT, $ZnCl_2$-CBZT and $ZnBr_2$-BZT.

		**$ZnCl_2$-BZT**	**$ZnCl_2$-CBZT**	**$ZnBr_2$-BZT**
100K	N-H···Cl	2.312 Å, 2.441 Å	2.325 Å	2.532 Å, 2.659 Å
	C-H···Cl	2.832 Å	2.928 Å	3.015 Å
	π–π	3.832 Å, 3.925 Å	3.550 Å, 3.688 Å	4.146 Å, 4.134 Å
297K	N-H···Cl	2.258 Å, 2.345 Å	2.360 Å	2.577 Å, 2.698 Å
	C-H···Cl	2.913 Å	3.015 Å	3.045 Å
	π–π	3.901 Å, 4.056 Å	3.611 Å, 3.812 Å	4.245 Å, 3.970 Å

3. Tunable fluorescence and RTP of the metal-organic halide microcrystals

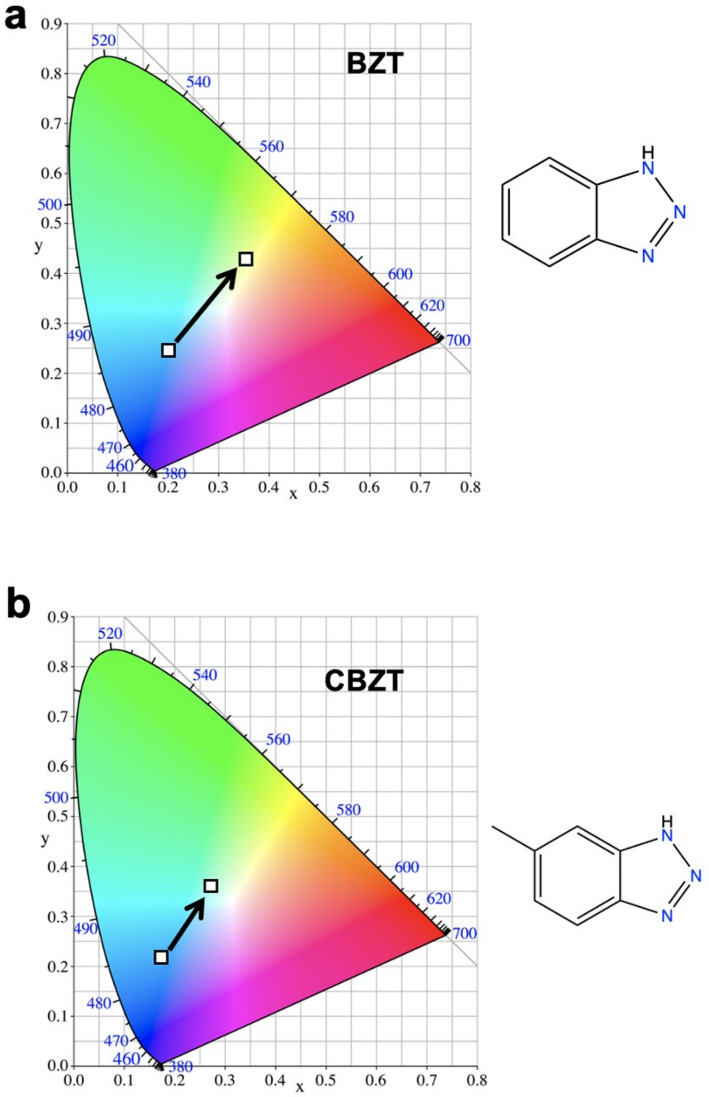

Fig. S4 The prompt and delayed emission positions of the pristine ligands BZT (469 nm and 529 nm) and CBZT (467 nm and 477 nm) are close to each other, indicating incomplete separation between fluorescence and phosphorescence. The corresponding positions of prompt and delayed emission in CIE chromaticity coordinates for organic ligands BZT (a) and CBZT (b).

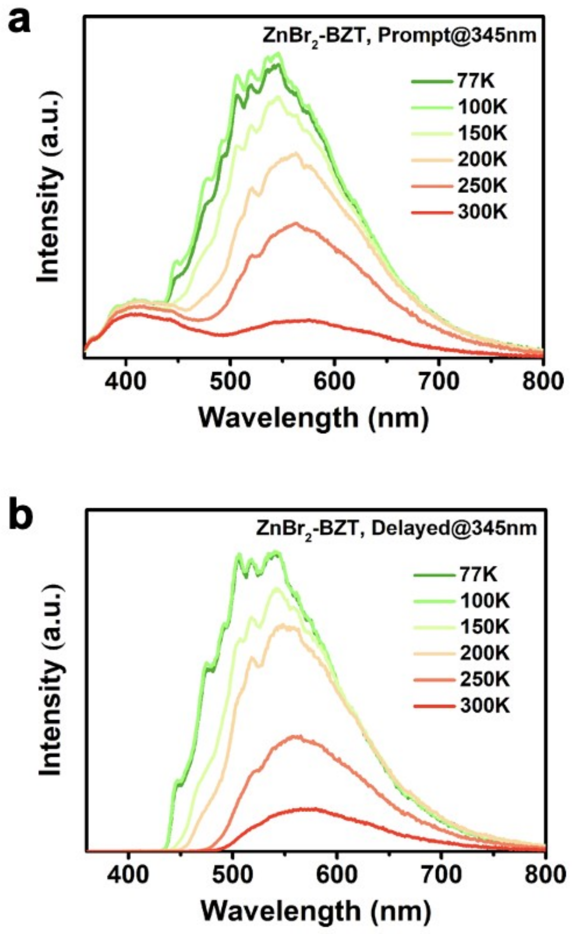

Fig. S5 Temperature-dependent prompt spectra present that the emission intensities of $ZnBr_2$-BZT at 408 nm gradually increase as temperature rises, suggesting that the energy levels at 408 nm is thermally activated. With increasing temperature, the emission intensities of delayed spectra for $ZnBr_2$-BZT gradually decrease, and the corresponding emission peaks appear red-shifted.

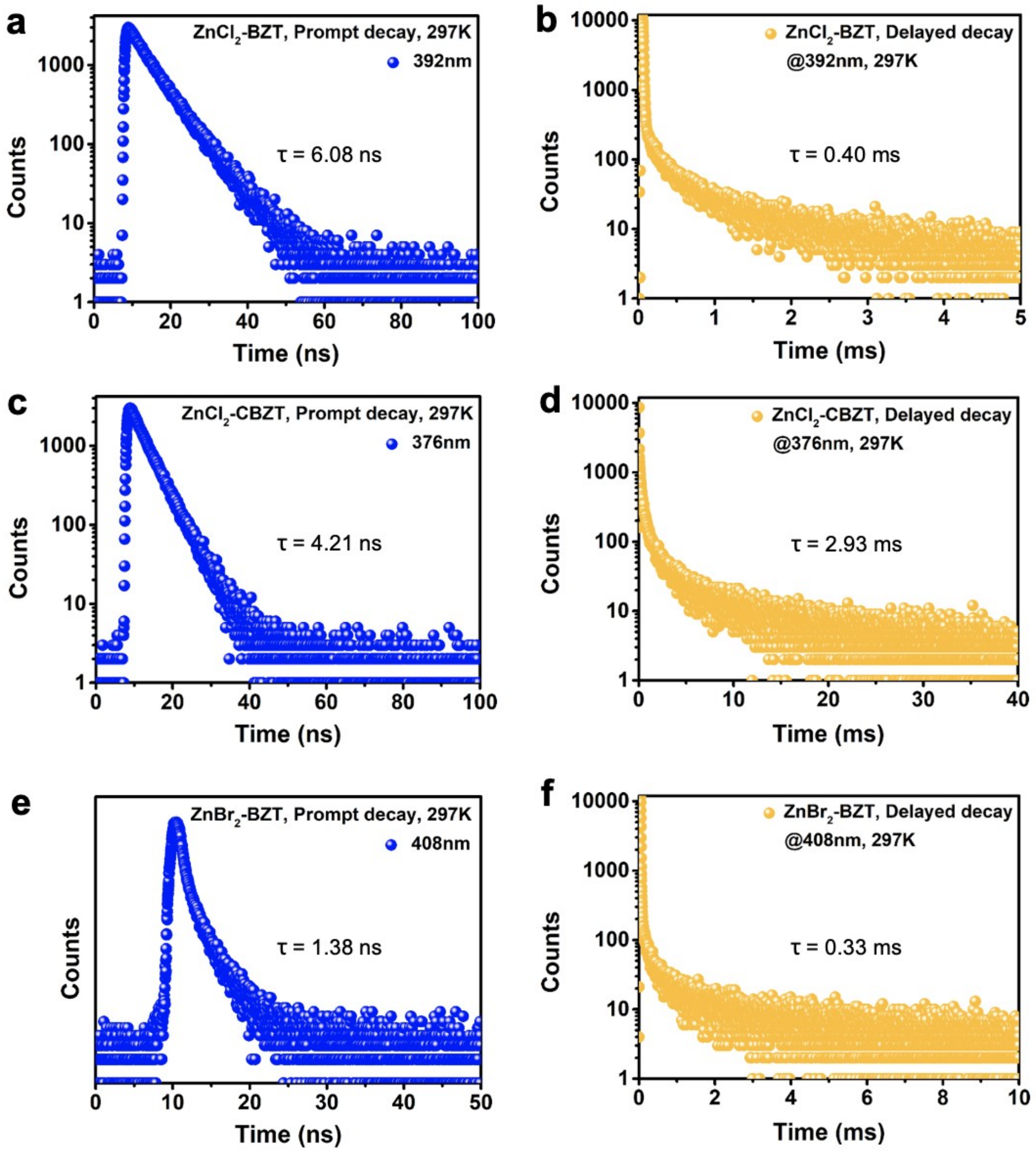

Fig. S6 The time-resolved prompt and delayed decay of $ZnCl_2$-BZT at 392 nm, $ZnCl_2$-CBZT at 376 nm and $ZnBr_2$-BZT at 408 nm. The main emission peaks of these prompt spectra simultaneously display short-lived (nanosecond scale) and long-lived (microsecond scale) lifetimes, proving that these emissions feature typical TADF characteristics through the reverse ISC (RISC) process between triplet and singlet excited states.

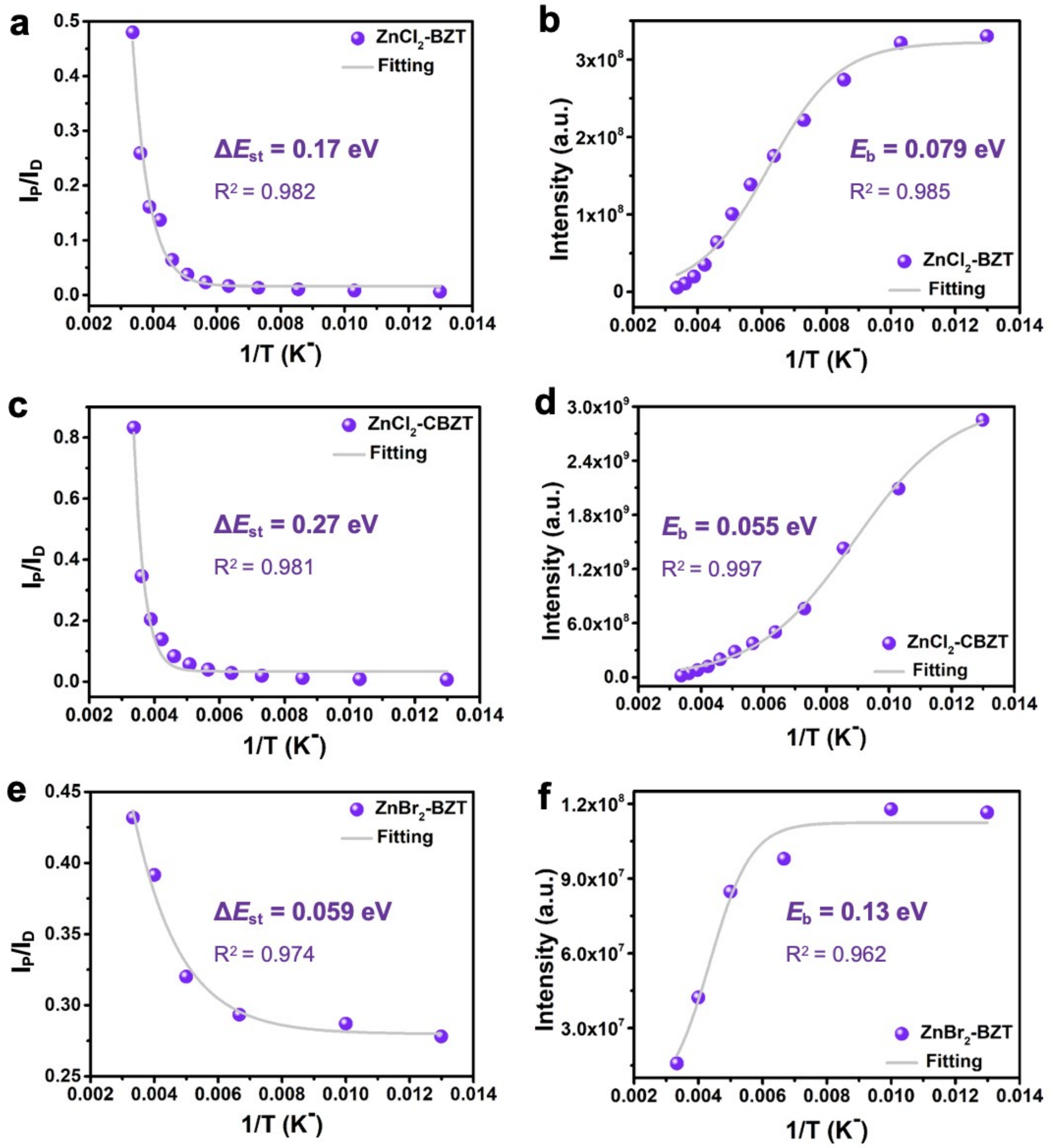

Fig. S7 The integrated intensity ratio between prompt and delayed spectra (a, c, e) as well as the Arrhenius plot of integrated intensity of the delayed spectra versus the reciprocal of temperature (1/T) (b, d, f).

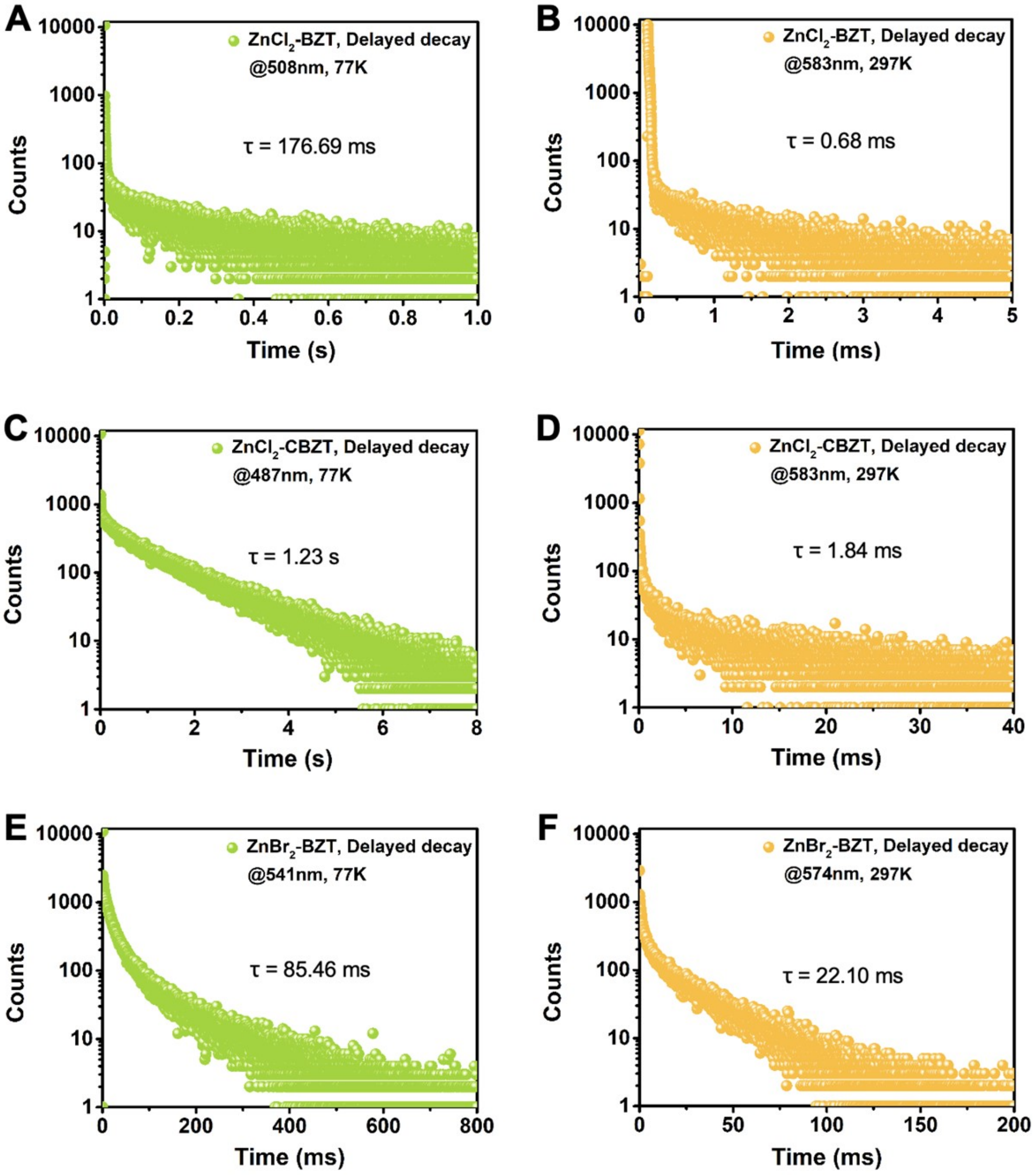

Fig. S8 The phosphorescence decay of $ZnCl_2$-BZT (a, b), $ZnCl_2$-CBZT (c, d), and $ZnBr_2$-BZT (e, f) at 77 K and 297 K, which exhibit the phosphorescence lifetimes of the dominant emission peaks for three metal-organic halide microcrystals decrease greatly as temperature rising from 77 K to 297 K.

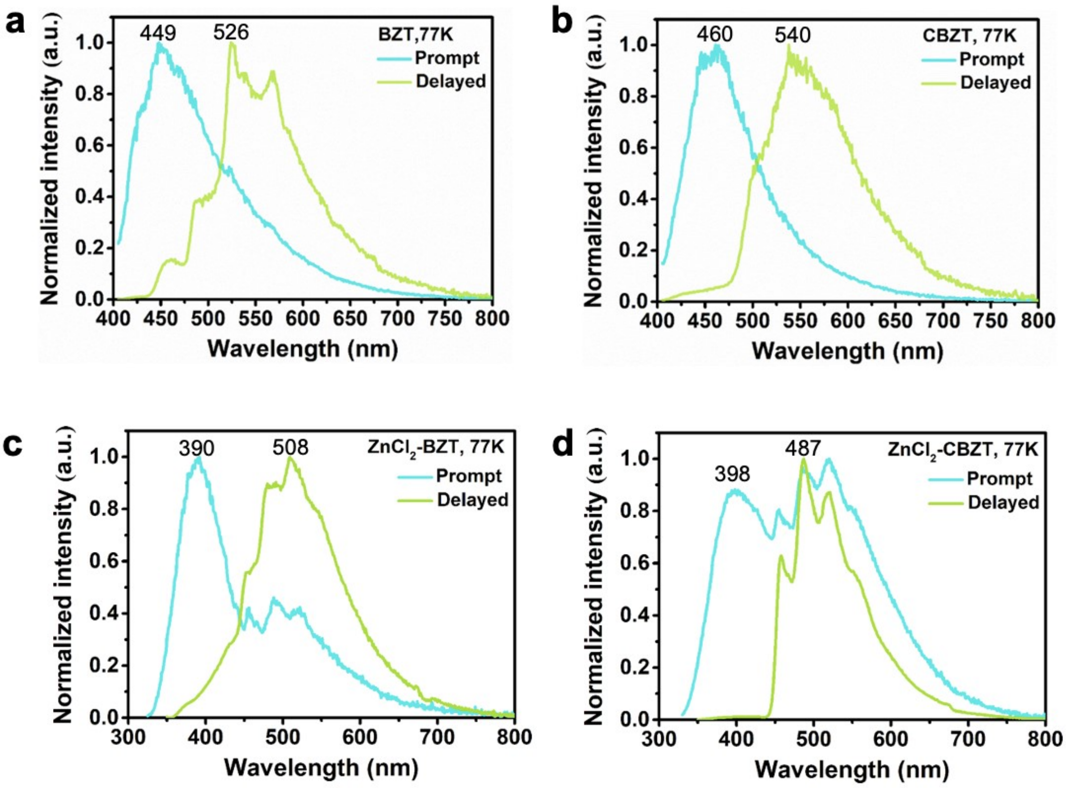

Fig. S9 The prompt and delayed spectra of BZT (a), CBZT (b), $ZnCl_2$-BZT (c), $ZnCl_2$-CBZT (d) at 77 K.

Table S4. The delayed lifetimes of dominant emission peaks for $ZnCl_2$-BZT, $ZnCl_2$-CBZT and $ZnBr_2$-BZT at 77 K and 297 K.

	**$ZnCl_2$-BZT**	**$ZnCl_2$-CBZT**	**$ZnBr_2$-BZT**
77 K	176.69 ms (508 nm)	1.23 s (487 nm)	85.46 ms (541 nm)
297 K	0.68 ms (583 nm)	1.84 ms (583 nm)	22.10 ms (574 nm)

4. **Mechanism on the light-emitting metal-organic halide microcrystals**

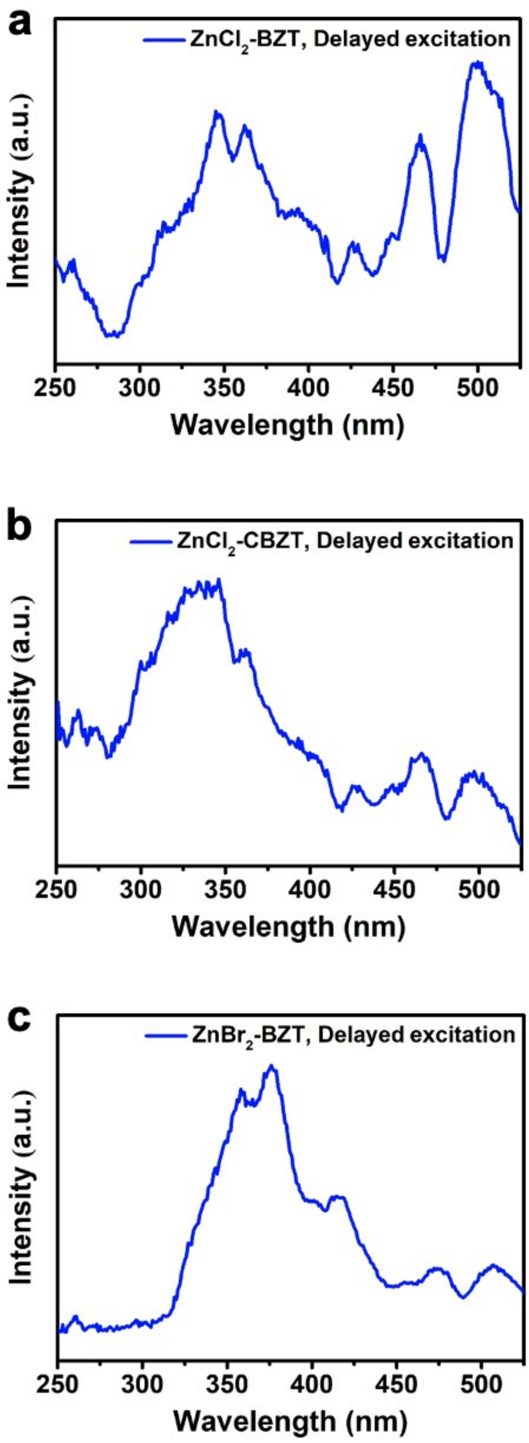

Fig. S10 Excitation peaks ranging from 400 nm to 525 nm in the delayed excitation spectra under the condition of the emission of 550 nm, which elucidate that these intermediate triplet excited states are highly excitable.

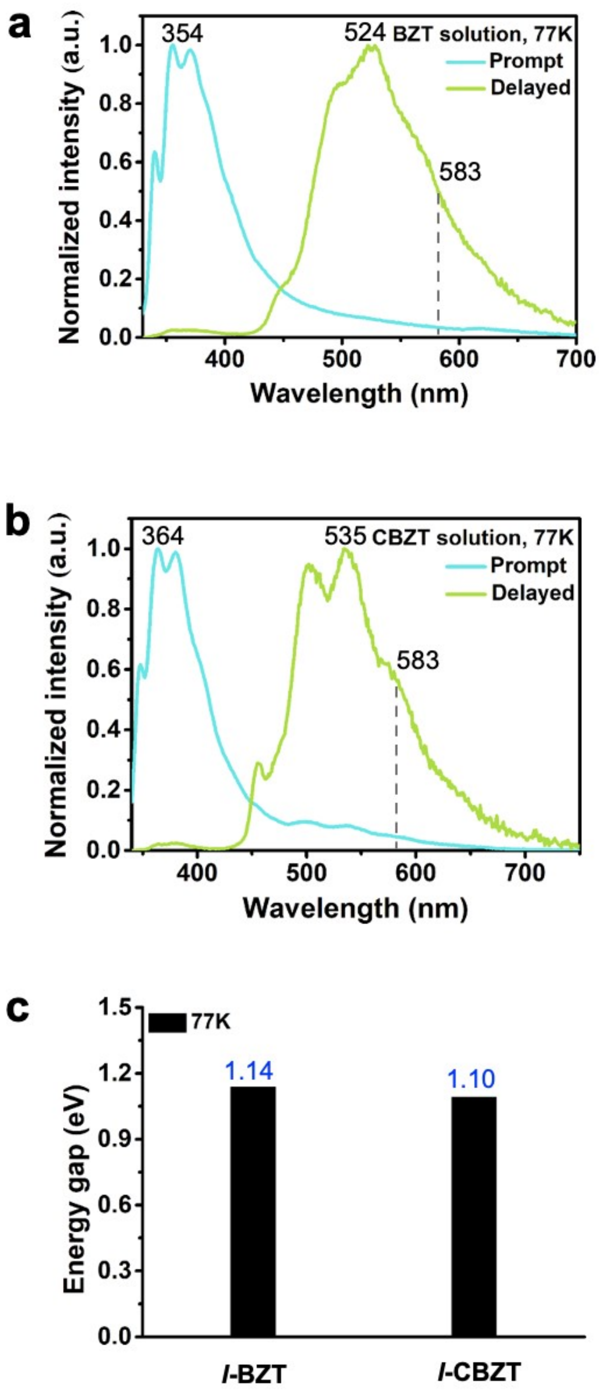

Fig. S11 The comparison of prompt and delayed spectra for BZT (a) and CBZT (b) solutions, as well as the calculated differences between dominant emission peaks (c) of the pristine ligand solutions are displayed.

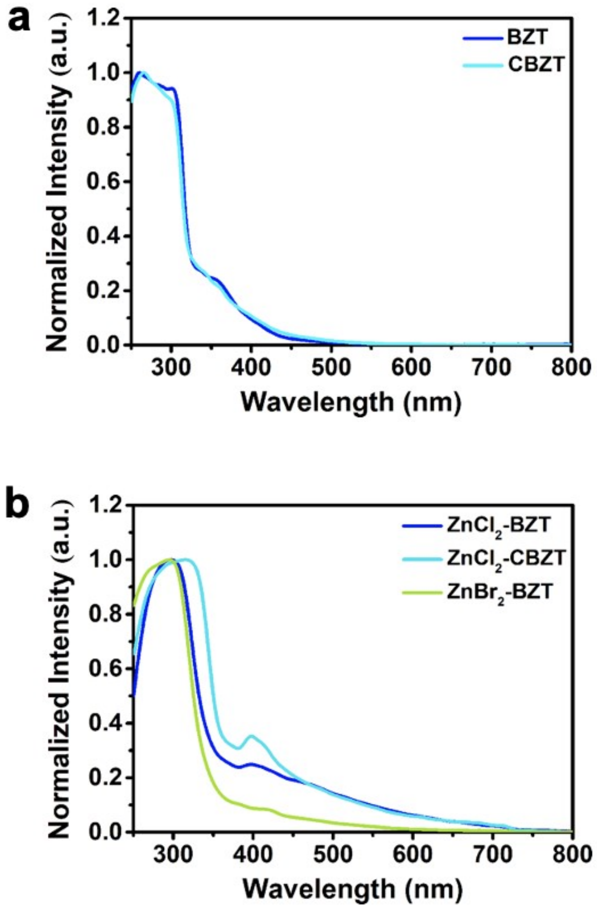

Fig. S12 Solid-state UV-vis absorption spectra of organic ligands BZT and CBZT (a), as well as the as-prepared metal-organic halide microcrystals under ambient conditions $ZnCl_2$-BZT, $ZnCl_2$-CBZT, and $ZnBr_2$-BZT (b).

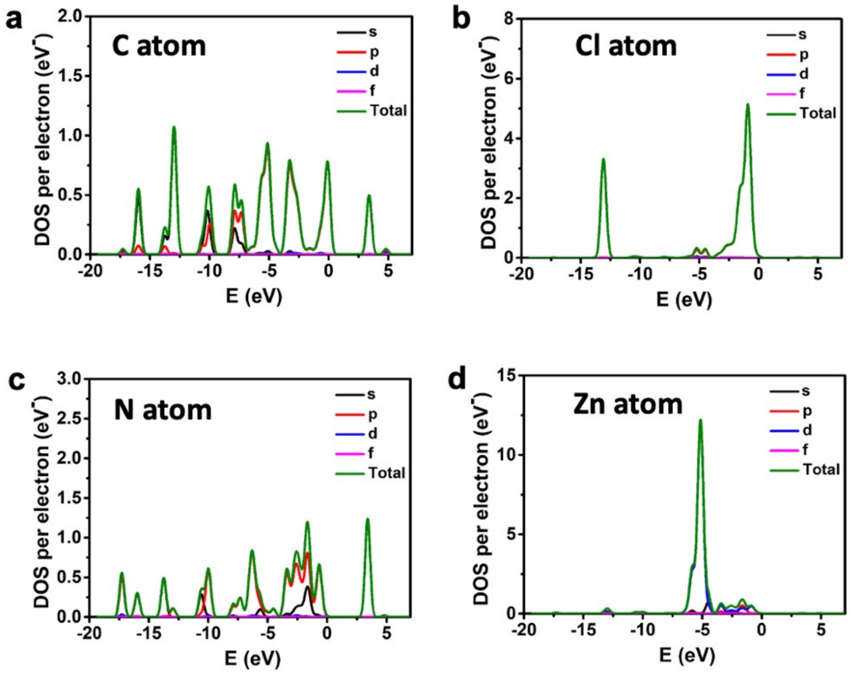

Fig. S13 The PDOS of the atoms in metal-organic halide microcrystal $ZnCl_2$-BZT. These data prove that the valence bands (VB) originate from the *p* orbitals of halogen ions, while the conduction bands (CB) derive from the *p* orbitals of C and N atoms.

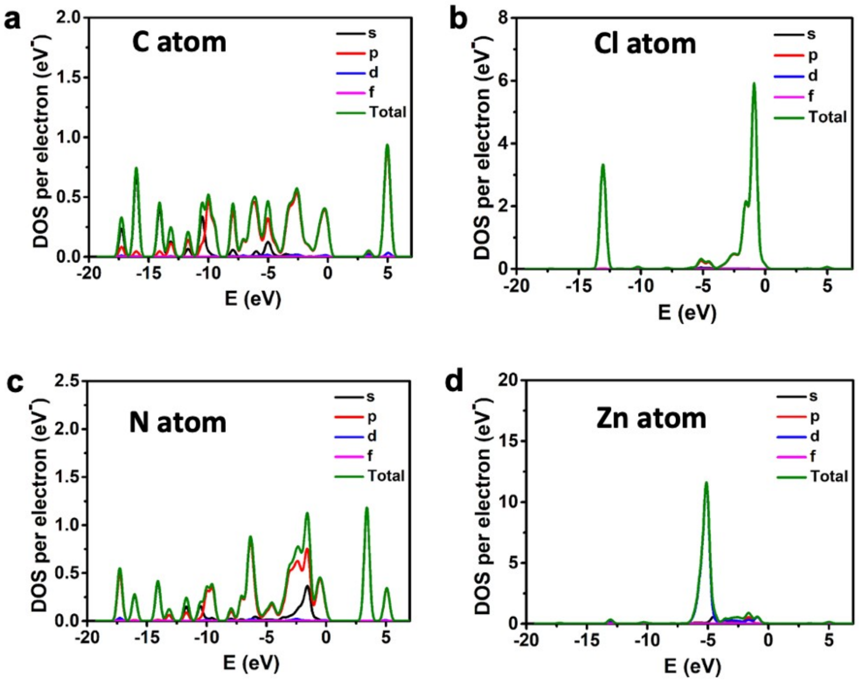

Fig. S14 The PDOS of the atoms in metal-organic halide microcrystal $ZnCl_2$-CBZT. These data prove that the valence bands (VB) originate from the *p* orbitals of halogen ions, while the conduction bands (CB) derive from the *p* orbitals of C and N atoms.

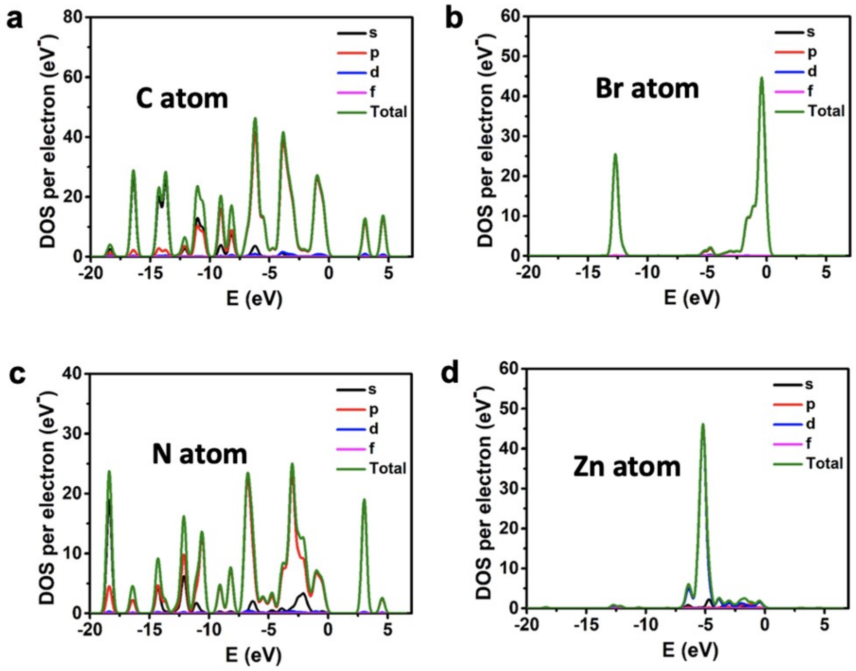

Fig. S15 These data prove that the valence bands (VB) originate from the *p* orbitals of halogen ions, while the conduction bands (CB) derive from the *p* orbitals of C and N atoms.

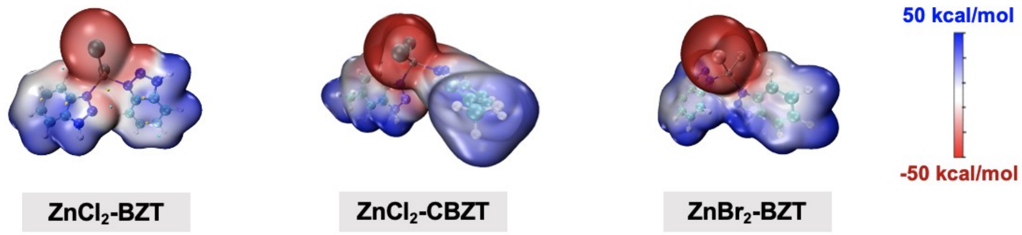

Fig. S16 Electrostatic potential (ESP) difference for metal-organic halide microcrystals $ZnCl_2$-BZT, $ZnCl_2$-CBZT, and $ZnBr_2$-BZT. ESP calculations reveal the respective electron density distributions (red color area: higher electron density; blue color area: lower electron density), which highlight the trend in charge transfer from halogen ions to organic ligands.

Table S5. The comparison of electrostatic potentials for $ZnCl_2$-BZT, $ZnCl_2$-CBZT and $ZnBr_2$-BZT.

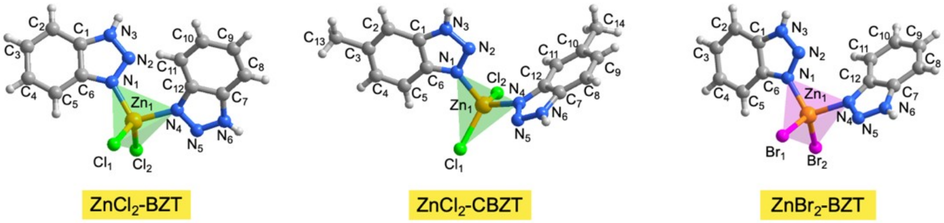

Atom	$ZnCl_2$-BZT ESP (kcal/mol)	$ZnCl_2$-CBZT ESP (kcal/mol)	Atom	$ZnBr_2$-BZT ESP (kcal/mol)
Zn_1	-26.03	-24.41	Zn_1	-31.16
Cl_1	-51.52	-50.20	Br_1	-42.67
Cl_2	-47.37	-46.84	Br_2	-39.43
N_1	-3.71	-7.52	N_1	-6.51
N_2	-1.34	-4.65	N_2	-0.92
N_3	23.24	15.34	N_3	21.73
N_4	-8.71	-8.91	N_4	-8.88
N_5	-23.25	-17.00	N_5	-19.34
N_6	13.60	15.02	N_6	14.69
C_1	17.63	11.03	C_1	15.56
C_2	5.86	-0.74	C_2	3.29
C_3	10.78	5.81	C_3	9.07
C_4	-1.79	-6.92	C_4	-3.65
C_5	6.05	3.56	C_5	4.31
C_6	1.87	-2.14	C_6	0.13
C_7	13.09	12.19	C_7	13.12
C_8	3.33	1.83	C_8	3.95

C_9	8.73	7.18	C_9	8.68
C_{10}	1.59	-3.35	C_{10}	2.19
C_{11}	5.88	4.26	C_{11}	5.78
C_{12}	4.52	1.79	C_{12}	4.58

5. Temperature-gating photonic communication

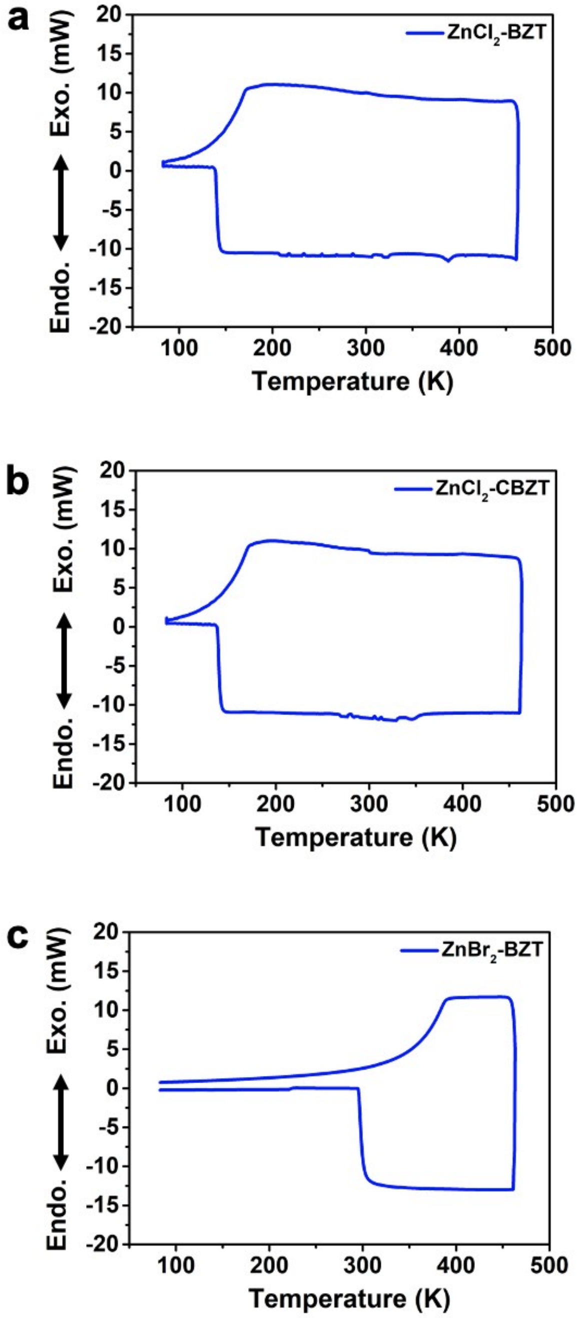

Fig. S17 DSC curves for metal-organic halide microcrystals $ZnCl_2$-BZT, $ZnCl_2$-CBZT and $ZnBr_2$-BZT. The crystal structures of metal-organic halides are highly stable in the temperature range of 83 K to 463 K, covering the extreme surface temperatures of Earth, Mars, and the moon.

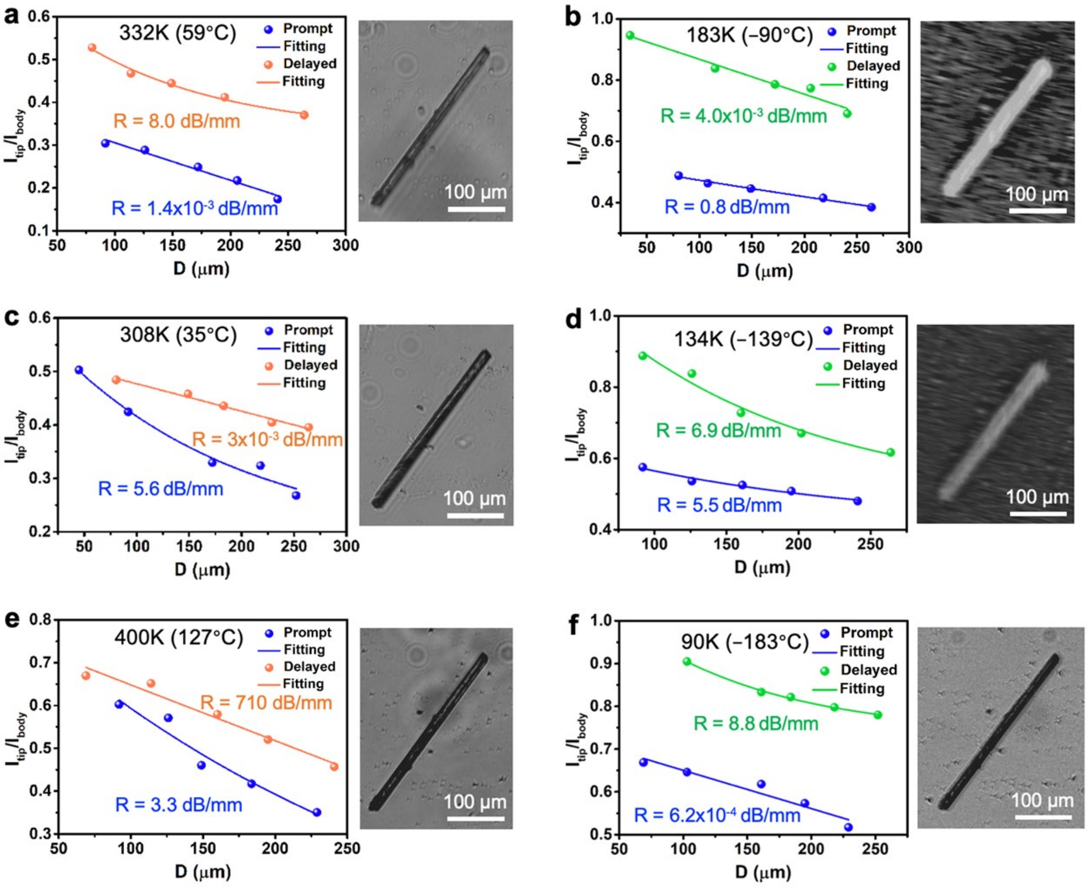

Fig. S18 The calculation of optical loss coefficient R. The ratios of the intensity I_{tip}/I_{body} against the distance D and the corresponding images in bright field under the simulated extreme surface temperatures on Earth (332 K and 183 K) (a, b), Mars (308 K and 134 K) (c, d), and Moon (400 K and 90 K) (e, f), and 1 dB/μm = 10^3 dB/mm.

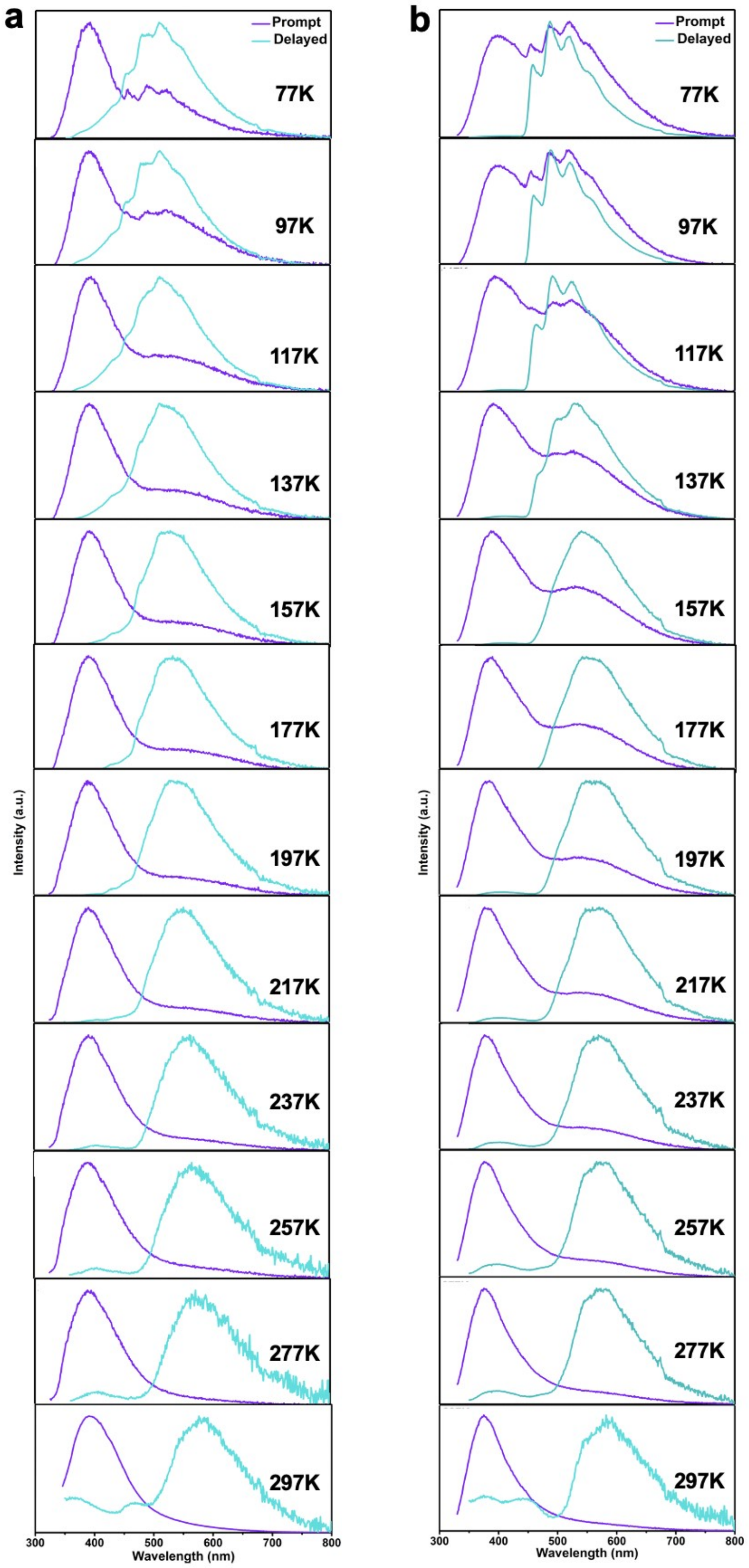

Fig. S19 Temperature-dependent prompt and delayed spectra: fluorescence and phosphorescence predominate in the prompt and delayed emission regions, respectively. (a) $ZnCl_2$-BZT, (b) $ZnCl_2$-CBZT.

Table S6. The comparison of the optical loss coefficient in *ref.*[10-21] and this work.

Name	Molecule structure	OLC/ dB mm^{-1}	Reference
**$ZnCl_2$-BZT**	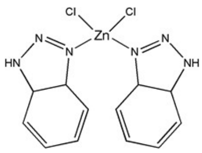	6.2x10^{-4}	**this work**
**DPIN**	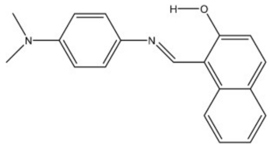	0.270	**10**
**Polymorph A**	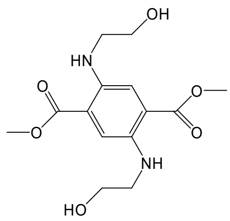	0.351	**11**
**PDI**	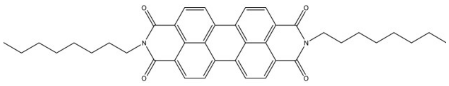	130	**12**
**Zn-Bpeb**	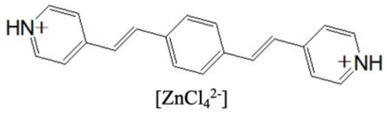	0.839	**13**
**1d@2d**	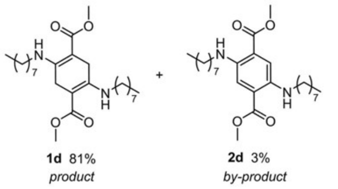	0.272<br>0.196	**14**
**DPEpe-HCl**	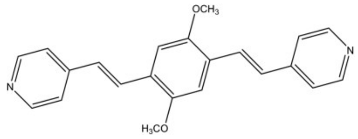	18.1	**15**
**BCZ**	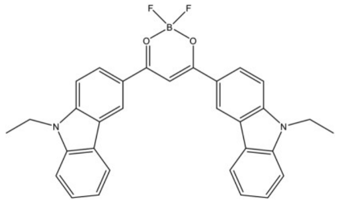	33	**16**

**C1**	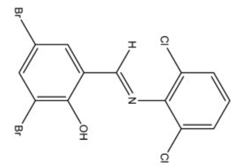	9.43	**17**
**DCF**	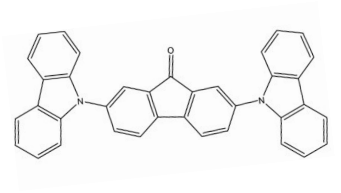	23.9	**18**
**Eu-BTC**	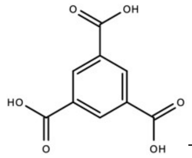 + Eu^{2+}	12	**19**
**FTCs**	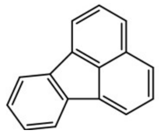 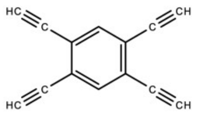	18.1	**20**
**9AC-TCNB**<br>**(1D)**	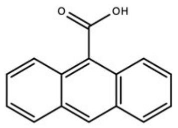 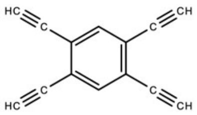	6.71<br>6.60	**21**

6. Supporting references

1. B. Delley, *J. Chem. Phys.,* 1990, **92**, 508.
2. B. Delley, *J. Chem. Phys.,* 2000, **113**, 7756.
3. J. P. Perdew, J. A. Chevary, S. H. Vosko, K. A. Jackson, M. R. Pederson, D. J. Singh and C. Fiolhais, *Phys. Rev. B.,* 1992, **46**, 6671.
4. M. J. Frisch, G. W. Trucks, H. B. Schlegel, G. E. Scuseria, M. A. Robb, J. R. Cheeseman, G. Scalmani, V. Barone, B. Mennucci and G. A. Petersson, *Gaussian 09, Revision D.01* (Gaussian Inc., 2009).
5. M. E. Casida, C. Jamorski, K. C. Casida and D. R. Salahub, *J. Chem. Phys.,* 1998, **108**, 4439.
6. R. E. Stratmann, G. E. Scuseria and M. J. Frisch, *J. Chem. Phys.,* 1998, **109**, 8218.
7. M. Kone, B. Illien, J. Graton and C. Laurence, *J. Phys. Chem.,* 2005, **109**, 11907.
8. T. Lu and F. Chen, *J. Comput. Chem.,* 2012, **33**, 580.
9. W. Humphrey, A. Dalke and K. Schulten, *J. Mol. Graph.,* 1996, **14**, 33.
10. H. Liu, Z. Lu, Z. Zhang, Y. Wang and H. Zhang, *Angew. Chem. Int. Ed.,* 2018, **57**, 8448.
11. B. Liu, Q. Di, W. Liu, C. Wang, Y. Wang and H. Zhang, *J. Phys. Chem. Lett.,* 2019, **10**, 1437.
12. Q. Bao, B. M. Goh, B. Yan, T. Yu, Z. Shen and K. P. Loh, *Adv. Mater.,* 2010, **22**, 3661.
13. S. Wu, B. Zhou and D. Yan, *ACS Appl. Mater. Interfaces,* 2021, **13**, 26451.
14. R. Huang, C. Wang, Y. Wang and H. Zhang, *Adv. Mater.,* 2018, **30**, 1800814.
15. M. P. Zhuo, X. Y. Fei, Y. C. Tao, J. Fan, X. D. Wang, W. F. Xie and L. S. Liao, *ACS Appl. Mater. Interfaces,* 2019, **11**, 5298.
16. P. Z. Chen, H. Zhang, L. Y. Niu, Y. Zhang, Y. Z. Chen, H. B. Fu and Q. Z. Yang, *Adv. Funct. Mater.,* 2017, **27**, 1700332.

17. M. Annadhasan, D. P. Karothu, R. Chinnasamy, L. Catalano, E. Ahmed, S. Ghosh, P. Naumov and R. Chandrasekar, *Angew. Chem. Int. Ed.,* 2020, **59**, 13821.
18. Q. Li, W. Jin, M. Chu, W. Zhang, J. Gu, B. Shahid, A. Chen, Y. Yu, S. Qiao and Y. S. Zhao, *Nanoscale.,* 2018, **10**, 4680.
19. X. Yang, X. Lin, Y. Zhao, Y. S. Zhao and D. Yan, *Angew. Chem. Int. Ed.,* 2017, **56**, 7853.
20. X. Ye, Y. Liu, Q. Guo, Q. Han, C. Ge, S. Cui, L. Zhang and X. Tao, *Nat. Commun*., 2019, **10**, 761.
21. J. Wang, S. Zhang, S. Xu, A. Li, B. Li, L. Ye, Y. Geng, Y. Tian and W. Xu, *Adv. Optical. Mater.,* 2019, **8**, 1901280.
